# Supplementary material for: Click-electrochemistry for the rapid labeling of virus, bacteria and cell surfaces
Source: Nat Commun. 2023 Aug 23;14:5122. doi: 10.1038/s41467-023-40534-0 (PMC10447534; doi:10.1038/s41467-023-40534-0)
Supplement: Supplementary file 1 — Supplementary Information [file 41467_2023_40534_MOESM1_ESM.pdf]

Click-electrochemistry for the rapid labeling of virus, bacteria and cell surfaces

Supplementary Information

Authors: Sébastien Depienne,[1] Mohammed Bouzelha,[2] Emmanuelle Courtois,[3] Karine Pavageau,[2] Pierre-Alban Lalys,[1] Maia Marchand,[1] Dimitri Alvarez-Dorta,[1,4] Steven Nedellec,[5] Laura Marín-Fernández,[3] Cyrille Grandjean,[3] Mohammed Boujtita,[1] David Deniaud,[1] Mathieu Mével,[2*] and Sébastien G. Gouin.[1*]

Affiliations:
[1]Nantes Université, CNRS, CEISAM UMR 6230, F-44000 Nantes, France
[2]Nantes Université, TaRGeT, Translational Research for Gene Therapies, CHU Nantes, INSERM, UMR 1089, F-44000 Nantes, France
[3]Nantes Université, CNRS, US2B, UMR 6286, F-44000 Nantes, France
[4]Capacités, 26 Bd Vincent Gâche, 44200 Nantes
[5]Nantes Université, CHU Nantes, CNRS, Inserm, BioCore, US16, SFR Bonamy, Nantes, France
Corresponding authors: mathieu.mevel@univ-nantes.fr; sebastien.gouin@univ-nantes.fr

Table of contents

Generalities ... 2

Organic synthesis ... 2

Electrobioconjugation ... 3

I. Synthesis and characterization of luminol anchors ... 5

1) Azide derivative ... 5

2) Carbohydrate derivatives ... 6

3) Biotin derivative ... 11

II. eY-click on AAV2: Procedures and characterizations ... 13

Production and purification of AAV2 ... 13

Electrobioconjugation procedure (*1 mL scale setup*) ... 13

Characterizations ... 13

III. eY-click on bacterial strains: Procedures and characterizations ... 17

Electrobioconjugation procedure (*5 mL scale setup*) ... 17

Characterizations ... 17

IV. eY-click on living cells: Procedures and characterizations ... 21

Electrobioconjugation procedure with azido derivative (*1 mL scale setup*) ... 21

Electrobioconjugation procedure with biotin derivative (*1 mL scale setup*) ... 21

Characterizations ... 22

V. NMR Spectra ... 26

VI. Uncropped Western Blots 31

VII. Representative gating strategy 39

VIII. Supplementary References ... 40

Generalities

Organic synthesis

Most of the chemical reagents were purchased from Sigma Aldrich®, Carbosynth®, Acros Organics®, Alfa Aesar® or TCI Chemical®. Reagents and solvents such as DCM, THF, Et_2O, TEA, Pyridine, MeOH, MeCN or DMF were purchased anhydrous from Sigma Aldrich®. All reagents were stored according to the detailed specifications and used without further purification. Reactions requiring anhydrous conditions were performed under positive nitrogen or argon pressure. Usual reaction monitoring was carried out with thin layer chromatography (TLC) on Merck 60 F_{254} silica gel plates. Revelations were performed under UV light (254 nm) or by dipping in a solution of cerium molybdate, potassium permanganate, sulfuric acid or vanillin and subsequently heated. Purification by silica gel chromatography were carried on Silica 60 M 0.04 – 0.063 mm.

^{1}H and ^{13}C NMR were recorded on Bruker Avance 300 or Bruker Avance 400 spectrometers. NMR spectra were assigned on the basis of the following 1D and 2D experiments: ^{1}H, ^{13}C, DEPT-135, COSY, HSCQ, HMBC and NOESY. All chemical shifts (δ) are shown in ppm on the X-axis using the residual solvent as internal standard (^{1}H NMR: $CDCl_3$ = 7.26 ppm, D_2O = 4.79 ppm, CD_3OD = 3.31 ppm, DMSO-d^6 = 2.50 ppm and ^{13}C NMR: $CDCl_3$ = 77.16 ppm, CD_3OD = 49.00 ppm, DMSO-d^6 = 39.52 ppm). Coupling constants (*J*) are reported in Hz and peak multiplicities are noted according to the following abbreviations: s = singlet, d = doublet, t = triplet, q = quartet, quin = quintet, m = multiplet, dd = doublet of doublet, dt = doublet of triplet, br = broad signal. Atom numbering used for NMR attribution is different from the numbers used in nomenclature of compounds. High-resolution mass spectrometry (HRMS) was recorded on a Waters Xevo GL-XS Qtof spectrometer coupled with an Acquity H-class LC apparatus. Ionization sources were performed with the available methods (ESI^+, ESI^-, $ASAP^+$, $ASAP^-$). A tolerance of 5 ppm was applied between calculated and experimental values.

Cell lines

The three cell lines HEK293, HUH7 and HeLa are not recorded in the misidentified cell lines list. They were cultured based on ATCC protocol. The HEK293 cell line comes from our Working Cell Bank (WCB) number 3 batch 14/03/2016 used routinely for AAV vector production in our vector core (https://umr1089.univ-nantes.fr/en/facilities-cores/cpv). They are regularly characterized by qPCR and RT-qPCR for E1A gene expression and they are tested for mycoplasma free and sterility. The HUH7 and HeLa cell lines were kindly provided by academic laboratories and are regularly tested for mycoplasma free and sterility. These regular characterizations are performed under our ISO 9001 quality management system (LRQA certificate number 10395603, expiry date 30 September 2024).

Electrobioconjugation

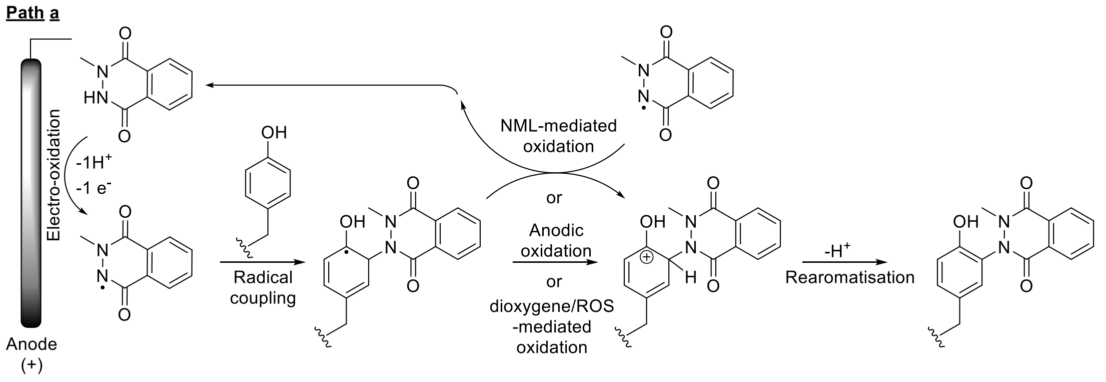

Supplementary Figure 1. Most likely path for eY-click with NML species
The precise oxidative radical coupling with tyrosines remains elusive, but is probably based on radical addition of the oxidized NML specie onto Y, followed by further oxidation of the eY-clicked product (by different pathways) and its spontaneous rearomatisation (path a).

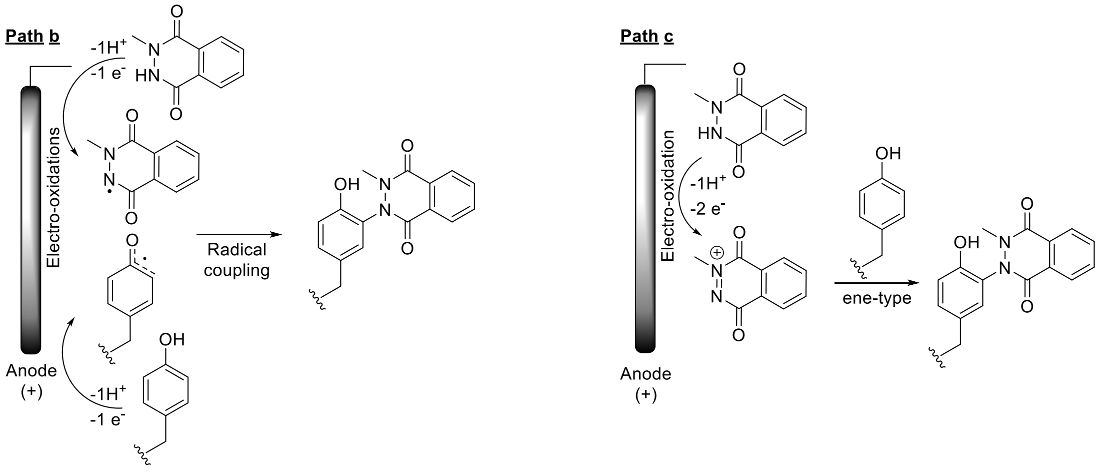

Supplementary Figure 2. Other possible paths for eY-click with NML species
No evidences definitively exclude tyrosine co-oxidation and radical co-ligation with oxidized NML (path b) or a two-electrons electro-oxidation of NML followed by ene-type addition of tyrosine in a similar manner than with PTAD (path c).
Aqueous buffers (PBS pH 7.4 and dPBS pH 7.4) were obtained from Sigma Aldrich®. Fluorescent cyclooctynes were purchased from Jena Biosciences®. SP-50 potentiostat was purchased from BioLogic®. Electrosynthesis equipments including ElectraSyn 2.0 (+ electrodes and vials) were purchased from IKA®.

Electro-bioconjugation experiments (eY-click) were performed with a three-electrode system using ElectraSyn 2.0 connected to SP-50 potentiostat for voltage control. All data were recorded using EC-Lab software. For all experiments, the three-electrode system was graphite plate as anode, platinum plate as cathode, and the reference was Ag/AgCl (a thin silver rod submerged with saturated aqueous KCl solution and protected from electrolysis mixture by a porous frit glass). Before each experiment, electrodes used were thoroughly washed with EtOH (SDS for AAV experiments) and distillated water, and working electrode was re-polished on high grit sand paper (<1200 grit) to prevent potential passivation. Depending on the biological target, experimental scale was varied between 5 mL and 1 mL with different dimensions of anode/cathode.

5 mL scale setup

Dimension of anode and cathode used was 4.0 cm x 0.8 cm x 0.15 cm (accessible surface = 7.72 cm^2). Considering a 5 mL solution with 90-95 % of electrodes dipping, the surface/volume ratio is estimated to be ~1.5 cm^2/mL. Electrobioconjugation experiments took place in the 5 mL ElectraSyn-adapted electrochemical setup (https://www.ika.com/fr/Produits-Lab-Eq/Kit-d%27electrochimie-csp-516/ElectraSyn-20-pro-Package-cpdt-40003261/) with electrical connections derivatized to SP-50 potentiostat with alligator clips.

1 mL scale setup

Dimension of anode and cathode used was 4.5 cm x 0.3 cm x 0.10 cm (accessible surface = 3.63 cm^2). Considering a 1 mL solution with 40-50 % of electrodes dipping, the surface/volume ratio is estimated to be ~1.5 cm^2/mL. As described in Figure S3, electrodes were connected to the electrodes holder part (**A**), the low-binding vial containing the solution of biological target and luminol derivative (**B**) was inserted into electrasyn-adapted 5 mL glass-vial (**C**) and the latter was screwed to the electrodes holder (**ABC**). Potentiostat was linked through electrical connections of the electrodes holder part and the resulting (**ABC**) vial was placed on the vial support on top of the orbital shaking device.

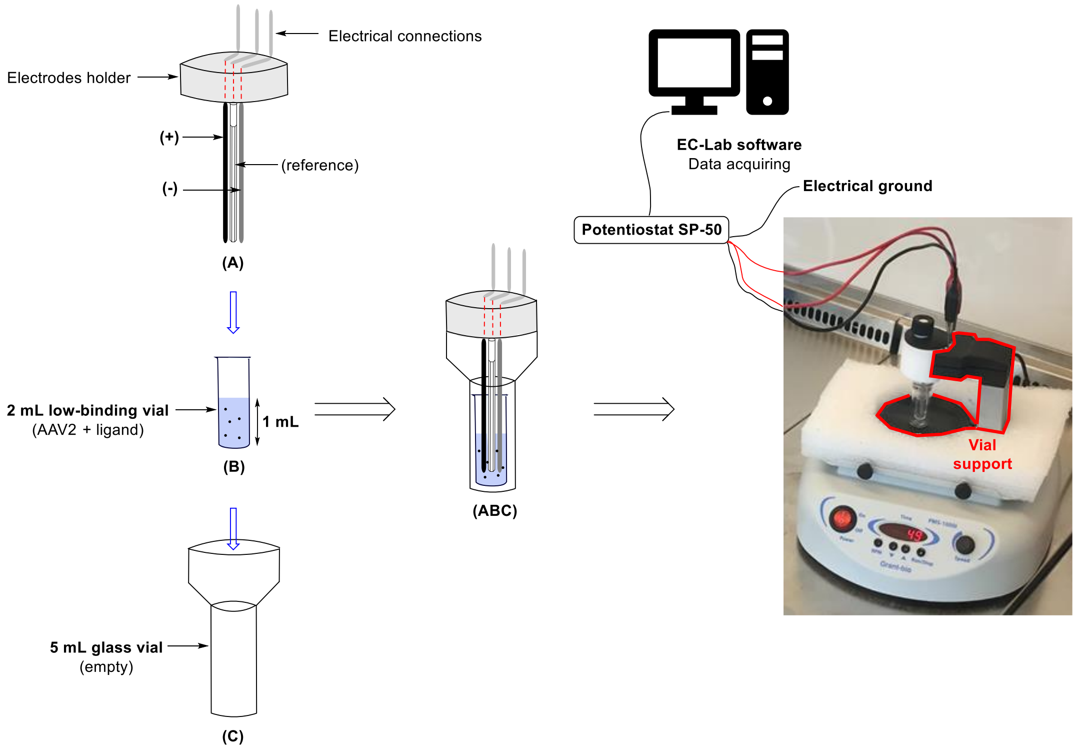

Supplementary Figure 3. **Experimental setup used for 1 mL scale electrobioconjugation**

I. Synthesis and characterization of luminol anchors

1) Azide derivative

➢ *Synthesis*

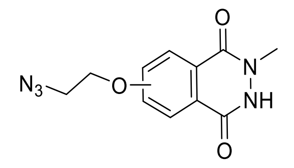

1
(Azide derivative)

Compound 1

Azide derivative was prepared in 5 steps as we previously reported.[1] **1** is formed as a mixture of 2 regioisomers in 60/40 proportions. ^{1}H NMR (400.16 MHz, DMSO-d^{6}, 298.15 K): δ_H 8.15 (d, *J*=8.8 Hz, 0.4H, H^{Ar}), 7.91 (d, *J*=8.8 Hz, 0.6H, H^{Ar}), 7.62 (d, *J*=2.7 Hz, 0.6H, H^{Ar}), 7.46 (dd, *J*=8.8 Hz, *J*=2.7 Hz, 0.6H, H^{Ar}), 7.43 (dd, *J*=8.8 Hz, *J*=2.7 Hz, 0.4H, H^{Ar}), 7.33 (d, *J*= 2.7 Hz, 0.4H, H^{Ar}), 4.36 (doubled t, 2H, OC*H$_2$*CH$_2$N), 3.72 (doubled t, 2H, OCH$_2$C*H$_2$*N), 3.55+3.53 (doubled s, 3H, NC*H$_3$*). ^{13}C NMR (100.62 MHz, DMSO-d^{6}, 298.15 K): δ_C 161.2, 160.8, 157.0, 156.9, 150.2, 149.9, 130.8, 128.7, 126.6, 122.7, 121.8, 121.0, 118.7, 108.3, 106.2, 67.4, 49.4, 37.6, 37.3. HRMS (ASAP^{-}): *m/z* calculated for $C_{11}H_{10}N_5O_3$ [M-H]$^{-}$ 260.0783 found 260.0784

➢ *Electrochemical behaviour*

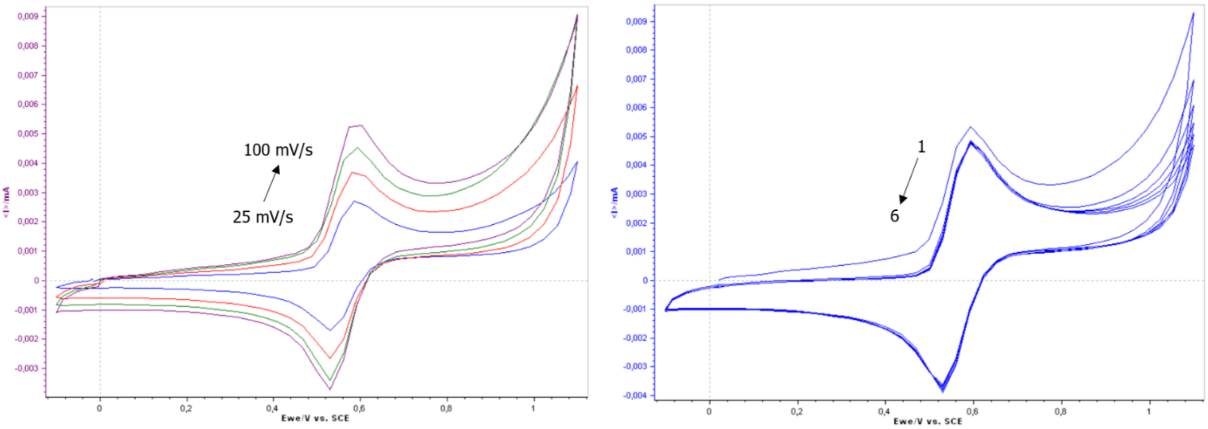

E_{ox} = 0.6 V *vs* SCE, reversible process

Supplementary Figure 4. Electrochemical behaviour of azide derivative 1
1 (1 mM) in MeCN/PB 50 mM pH 7.4 recorded at a 2 mm disc cathode: glassy carbon electrode, anode: platinum wire, reference: saturated calomel electrode. **(left)** Cyclic voltammetry at scan rate: 25, 50, 75 and 100 mV/s. **(right)** Multicyclic voltammetry (n= 6) at 100 mV/s

2) Carbohydrate derivatives

➢ *Synthesis*

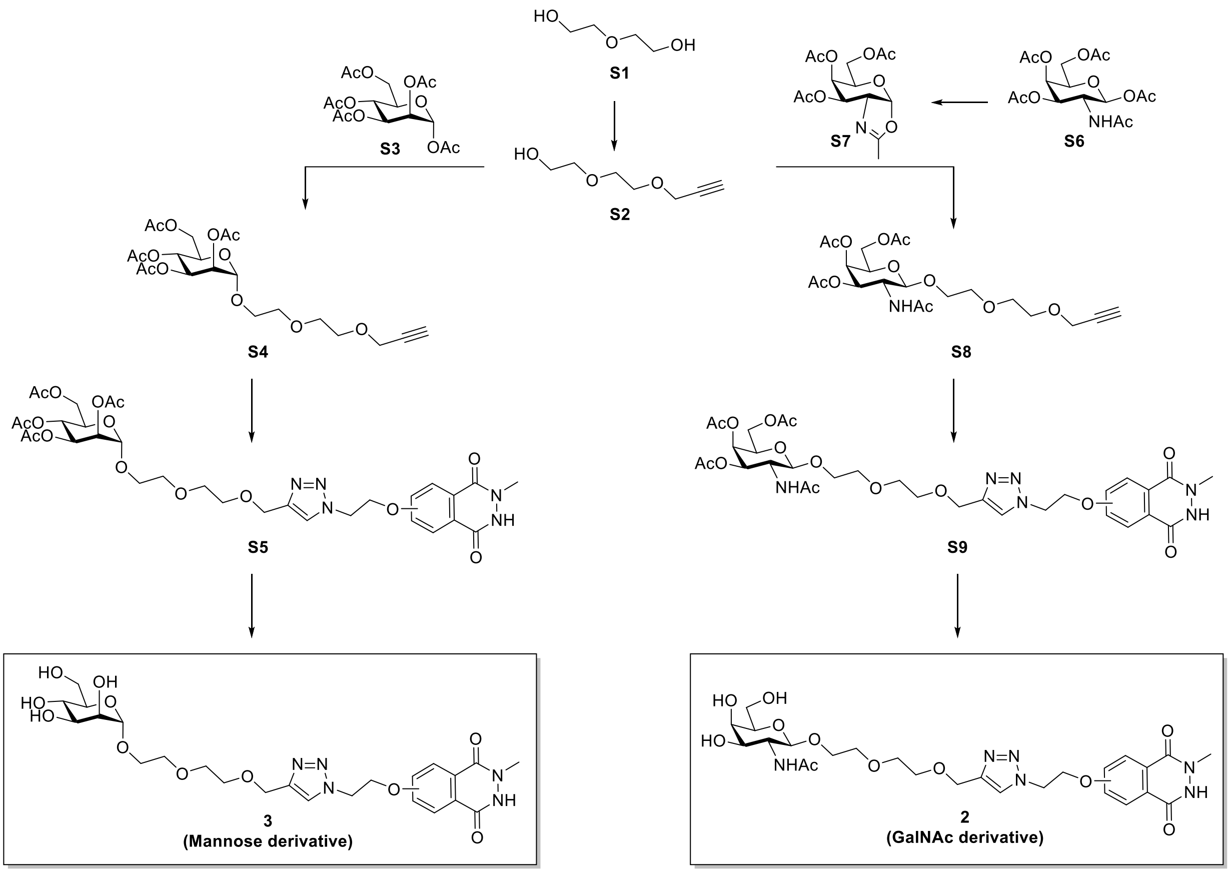

Supplementary Figure 5. **Synthesis of Mannose derivative and GalNAc derivative**

Compound S2

To a suspension of commercially available tBuOK (793 mg, 7.07 mmol, 1 equiv.) in anhydrous THF (25 mL), commercially available diethylene glycol **S1** (1.34 mL, 14.13 mmol, 2 equiv.) was added at 0 °C and under positive nitrogen atmosphere. Mixture was stirred at room temperature during 30 min. 80% propargyl bromide solution in toluene (0.61 mL, 7.07 mmol, 1 equiv.) was then solubilized in 5 mL of anhydrous THF and added dropwise to the mixture. Reaction was stirred at room temperature during 18 h (completion monitored by TLC, Rf = 0.3 in pure AcOEt) and mixture was filtered on a celite pad washed with THF. The obtained solution was concentrated under reduced pressure and purified by silica gel chromatography (2:8 CyHex/AcOEt to 100% AcOEt) to afford **S2** (735 mg, 72 %) as a colorless oil. ^{1}H NMR (300.13 MHz, $CDCl_3$, 298.15 K): δ_H 4.20 (d, *J*=2.4 Hz, 2H, -C*H*$_2$C≡CH), 3.75-3.65 (m, 6H, H^{chain}), 3.60 (m, 2H, H^{chain}), 2.44 (t, *J*=2.4 Hz, 1H, -CH$_2$C≡C*H*) ; HRMS (ES$^+$): *m/z* calculated for $C_7H_{12}O_3Na$ [M+Na]$^+$ 167.0684 found 167.0689

Compound S4

To a solution of commercially available peracetylated mannose **S3** (1.7 g, 4.39 mmol, 1 equiv.) in anhydrous MeCN (50 mL, 0.1 M), at room temperature and under positive nitrogen atmosphere, was added mono-*O*-propargyl chain **3** (950 mg, 6.59 mmol, 1.5 equiv.). Mixture was cooled to 0 °C and $BF_3.OEt_2$ (1.6 mL, 13.18 mmol, 3 equiv.) was added dropwise. Reaction was stirred at room temperature during 48 h (completion monitored by TLC, Rf = 0.3 in 4:6 CyHex/AcOEt) and was quenched with a saturated solution of $NaHCO_3$. Aqueous layer was extracted three times with AcOEt and the combined organic layer was washed once with H_2O and once with brine before being dried over $MgSO_4$, filtered and concentrated under reduced pressure. The obtained crude compound was purified by silica gel chromatography (60:40 to 40:60 CyHex/AcOEt) to afford **5** (1.28 g, 63 %) as a colorless oil. ^{1}H NMR (300.13 MHz, $CDCl_3$, 298.15 K): δ_H 5.36 (dd, *J*=10.0 Hz, *J*=3.3 Hz, 1H, H^3), 5.32-5.24 (m, 2H, H^4 + H^2), 4.87 (d, *J*=1.6 Hz, 1H, H^1), 4.30 (dd, *J*=12.8 Hz, *J*=5.3 Hz, 1H, H^{6a}), 4.20 (d, *J*=2.4 Hz, 2H, CH_2C≡CH), 4.11-4.04 (m, 2H, H^5, H^{6b}), 3.87-3.77 (m, 1H, H^{chain}), 3.72-3.63 (m, 7H, H^{chain}), 2.44 (t, *J*=2.4 Hz, 1H, CH_2C≡C*H*), 2.15 (s, 3H, COCH_3), 2.09 (s, 3H, COCH_3), 2.03 (s, 3H, COCH_3), 1.98 (s, 3H, COCH_3) ; ^{13}C NMR (75.48 MHz, $CDCl_3$, 298.15 K): δ_C 170.8, 170.2, 170.0, 169.9, 97.9, 79.8, 74.7, 70.7, 70.2, 69.8, 69.3, 69.3, 68.6, 67.5, 66.3, 62.6, 58.6, 21.0, 20.9, 20.8, 20.8 ; HRMS (ES$^+$): *m/z* calculated for $C_{21}H_{30}O_{12}Na$ [M+Na]$^+$ 497.1635 found 497.1630

Compound S5

To a solution of **S4** (600 mg, 1.26 mmol, 1.1 equiv.) in dioxane (12 mL) were added prepared azide derivative **1** (300 mg, 1.15 mmol, 1 equiv.) and sodium ascorbate (273 mg, 1.38 mmol, 1.2 equiv.). An aqueous solution (3 mL) of $CuSO_4.H_2O$ (123 mg, 0.69 mmol, 0.6 equiv.) was then added and reaction was heated up to 70 °C and stirred protected from light with aluminium. After 45 min, completion was monitored by TLC (Rf = 0.3 in 9:1 DCM/MeOH) and mixture was cooled to room temperature. Chelex resin® was added (4 spatula) and mixture was left stirred 10 min. Resin was filtered off and washed twice with MeOH. The obtained solution was concentrated under reduced pressure and purified by silica gel chromatography (92:8 DCM/MeOH) to afford **S5** (750 mg, 89%) as a white solid. ^{1}H NMR (400.16 MHz, MeOD-d^4, 298.15 K): δ_H 8.17 (d, *J*=8.8 Hz, 0.6H, H^{Ar}), 8.12 (s, 1H, 1xC=C$H^{triazol}$), 7.94 (d, *J*=8.8 Hz, 0.4H, H^{Ar}), 7.67 (d, *J*=2.5 Hz, 0.4H, H^{Ar}), 7.42–7.35 (m, 1.6H, H^{Ar}), 5.28-5.20 (m, 3H, H^2 + H^3 + H^4), 4.94 – 4.89 (m, 2H, ArOCH_2CH_2N), 4.86 (d, *J*=1.3 Hz, 1H, H^1), 4.65 (s, 2H, H^{chain}) ; 4.63-4.58 (m, 2H, ArOCH_2CH_2N), 4.24–4.17 (m, 1H, H^{6a}), 4.13–4.05 (m, 2H, H^{6b} + H^5), 3.83–3.77 (m, 1H, H^7), 3.69–3.60 (m, 10H, H^7 + NCH_3 + 6xH^{chain}) ; ^{13}C NMR (100.62 MHz, MeOD-d^4, 298.15 K): δ_C 172.4, 171.6, 171.5, 171.5, 163.3, 162.8, 159.9, 159.8, 152.9, 152.3, 146.3, 131.9, 129.9,127.9, 125.9, 124.1, 123.4, 122.5, 109.5, 107.6, 98.9, 71.6, 71.2, 70.8, 70.8, 70.7, 69.8, 68.4, 68.2, 67.3, 65.1, 63.6, 50.8, 38.9, 38.7, 20.7 ; HRMS (ES$^-$): *m/z* calculated for $C_{32}H_{40}N_5O_{15}$ [M-H]$^-$ 734.2521 found 734.2522

Compound 3 (Mannose derivative)

To a solution of prepared **6** (100 mg, 0.14 mmol, 1 equiv.) in anhydrous 3:2 MeOH/THF (0.9 mL MeOH/0.6 mL THF, 0.1 M) a 1 M solution of MeONa in anhydrous MeOH (0.27 mL, 0.27 mmol, 2 equiv.) was added and reaction was left stirred at room temperature under nitrogen atmosphere. After 30 min, completion was monitored by TLC (Rf = 0-0.1 in 85:15 DCM/MeOH) and Dowex-50 acidic resin® (beforehand reactivated with conc. HCl and washed with water and MeOH) was added (4 spatula). Mixture was left stirred 15 min and resin was filtered off and washed twice with MeOH. The obtained solution was concentrated under reduced pressure and lyophilized to afford pure **7** (72 mg, 94%) as a white solid that

did not require further purification. ^1H NMR (400.16 MHz, DMSO-d^6, 298.15 K): δ_H 8.21+8.20 (doubled s, 1H, C=C$H^{triazol}$), 8.11 (d, J=8.7 Hz, 0.4H, H^{Ar}), 7.88 (d, J=8.7 Hz, 0.6H, H^{Ar}), 7.61 (d, J=2.5 Hz, 0.6H, H^{Ar}), 7.45-7.37 (2x dd, J=8.7 Hz, J=2.5 Hz, 1H, H^{Ar}), 7.31 (d, J=2.5 Hz, 0.4H, H^{Ar}), 4.82 (m, 2H, ArOCH_2CH$_2$N), 4.65-4.57 (m, 3H, H^1 + ArOCH$_2$CH_2N), 4.53 (s, 2H, OCH_2C^{triazol}), 3.69-3.28 (m, 17H, 6x ^{carb} + 8H^{chain} + NCH_3) ; ^{13}C NMR (100.62 MHz, DMSO-d^6, 298.15 K): δ_C 161.2, 160.7, 144.0, 130.7, 128.6, 126.6, 124.4, 122.7, 121.8, 121.1, 108.5, 99.99, 73.9, 70.9, 69.7, 69.5, 68.9, 66.9, 66.7, 63.5, 61.2, 48.8, 37.6 ; HRMS (ES$^-$): *m/z* calculated for $C_{24}H_{32}N_5O_{11}$ [M-H]$^-$ 566.2098 found 566.2100

Compound S7

To a solution of commercially available peracetylated galactosamine **S6** (2 g, 5.14 mmol, 1 equiv.) in anhydrous DCM (25 mL, 0.2 M) at 0 °C and under positive nitrogen atmosphere, TMSOTf (3.25 mL, 17.98 mmol, 3 equiv.) was added dropwise. The ice bath was removed and reaction was heated up to 50 °C and stirred during 5 h. Completion was monitored by TLC (Rf = 0.3 in 98:2 DCM/MeOH) and reaction was quenched with a saturated solution of $NaHCO_3$. Aqueous layer was extracted three time with DCM and the combined organic layer was washed once with H_2O and once with brine before being dried over $MgSO_4$, filtered and concentrated under reduced pressure. The obtained crude compound **S7** (1.65 g, orange oily solid) was used in next step without further purification.

Compound S8

To a solution of freshly prepared crude oxazoline **S7** (1.43 g, 4.34 mmol, 1.25 equiv.) in anhydrous DCM (30 mL, 0.1 M), at room temperature and under positive nitrogen atmosphere, mono-*O*-propargyl chain **S2** (500 mg, 3.47 mmol, 1 equiv.) was added. Mixture was cooled to 0 °C and TMSOTf (0.31 mL, 1.74 mmol, 0.5 equiv.) was added. After 24 h stirring at room temperature, completion was monitored by TLC (Rf = 0.25 in 95:5 DCM/MeOH) and reaction was quenched with a saturated solution of $NaHCO_3$. Aqueous layer was extracted once with DCM and the combined organic layer was washed once with H_2O and once with brine before being dried over $MgSO_4$, filtered and concentrated under reduced pressure. The obtained crude compound was purified by silica gel chromatography (95:5 DCM/MeOH) to afford **S8** (670 mg, 42 %) as a yellow oil. ^1H NMR (300.13 MHz, $CDCl_3$, 298.15 K): δ_H 6.46 (d, J=9.5 Hz, 1H, NHAc), 5.29 (m, 1H, H^4), 4.99 (dd, J=11.1 Hz, J=3.4 Hz, 1H, H^3), 4.82 (d, J=8.7 Hz, 1H, H^1), 4.34-4.21 (m, 3H, H^2 + -CH_2C≡CH), 4.14 (m, 2H, $H^{6a,b}$), 3.92-3.56 (m, 9H, H^5 + 8H^{chain}), 2.49 (t, J=2.4 Hz, 1H, -CH$_2$C≡CH), 2.14 (s, 3H, COCH_3), 2.03 (s, 3H, COCH_3), 1.97 (s, 3H, COCH_3), 1.95 (s, 3H, COCH_3) ; ^{13}C NMR (75.48 MHz, $CDCl_3$, 298.15 K): δ_C 170.6, 170.5, 170.5, 170.4, 102.5, 79.3, 75.4, 72.1, 71.1, 70.6, 70.1, 69.2, 68.2, 66.7, 61.7, 58.3, 50.5, 23.17, 20.8, 20.7, 20.7 ; HRMS (ES$^+$): *m/z* calculated for $C_{21}H_{31}NO_{11}Na$ [M+Na]$^+$ 496.1495 found 496.1797

Compound S9

To a solution of prepared **S8** (585 mg, 1.24 mmol, 1.1 equiv.) in dioxane (9.5 mL, final conc. 0.1 M) were added prepared azide derivative **1** (293 mg, 1.12 mmol, 1 equiv.) and sodium ascorbate (267 mg, 1.35 mmol, 1.2 equiv.). An aqueous solution (2.5 mL) of $CuSO_4.H_2O$ (120 mg, 0.67 mmol, 0.6 equiv.) was then added and reaction was heated up to 70 °C and stirred protected from light with aluminium. After 45 min, completion was monitored by TLC (Rf = 0.35 in 9:1 DCM/MeOH) and mixture was cooled to room temperature. Chelex resin® was added (4 spatula) and mixture was left stirred 15 min. Resin was filtered off and washed twice with MeOH. The obtained solution was concentrated under reduced pressure and purified by silica gel chromatography (93:7 DCM/MeOH) to afford **S9** (680 mg, 83%) as a white solid. ^1H NMR (400.16 MHz, DMSO-d^6, 298.15 K): δ_H 11.57 (*br* s, 1H, NH), 8.21+8.20 (doubled s, 1H, C=C$H^{triazol}$),

8.10 (d, *J*=8.7 Hz, 0.4H, H^{Ar}), 7.87 (d, *J*=8.7 Hz, 0.6H, H^{Ar}), 7.80 (d, *J*=9.2 Hz, 1H, N*H*Ac), 7.60 (d, *J*=2.5 Hz, 0.6H, H^{Ar}), 7.45-7.36 (2x dd, *J*=8.7 Hz, *J*=2.5 Hz, 1H, H^{Ar}), 7.30 (d, *J*=2.5 Hz, 0.4H, H^{Ar}), 5.21 (d, *J*=3.3 Hz, 1H, H^4), 4.97 (dd, *J*=11.2 Hz, *J*=3.4 Hz, 1H, H^3), 4.82 (m, 2H, ArOCH_2CH$_2$N), 4.60 (m, 2H, ArOCH$_2$CH_2N), 4.57-4.50 (d + s, *J*=8.7 Hz, 3H, H^1 + OCH_2C^{triazol}), 4.02 (m, 3H, H^5 + $H^{6a,b}$), 3.87 (m, 1H, H^2), 3.77 (m, 1H, H^{chain}), 3.61-3.45 (m, 10H, 7H^{chain} + NCH_3), 2.09 (s, 3H, COCH_3), 1.98 (s, 3H, COCH_3), 1.89 (s, 3H, COCH_3), 1.75 (s, 3H, COCH_3) ; ^{13}C NMR (100.62 MHz, DMSO-d^6, 298.15 K): δ_C 169.9, 169.8, 169.6, 169.3, 161.3, 160.7, 144.0, 130.7, 128.6, 126.6, 124.4, 122.8, 121.8, 121.1, 108.6, 100.9, 70.5, 69.9, 69.7, 69.4, 68.9, 68.2, 66.8, 66.7, 63.4, 61.4, 49.4, 48.8, 22.7, 20.5, 20.4, 20.4 ; HRMS (ES$^+$): *m/z* calculated for $C_{32}H_{43}N_6O_{14}$ [M+H]$^+$ 735.2837 found 735.2832

Compound 2 (GalNAc derivative)

To a solution of prepared **S9** (200 mg, 0.27 mmol, 1 equiv.) in anhydrous MeOH (3 mL, 0.1 M) was added a 1 M solution of MeONa in anhydrous MeOH (0.4 mL, 0.4 mmol, 1.5 equiv.) and reaction was left stirred at room temperature under nitrogen atmosphere. After 3 h, completion was monitored by TLC (Rf = 0-0.05 in 85:15 DCM/MeOH) and Dowex-50 acidic resin® (beforehand reactivated with conc. HCl and washed with water and MeOH) was added (4 spatula). Mixture was left stirred 15 min and resin was filtered off and washed twice with MeOH. The obtained solution was concentrated under reduced pressure and lyophilized to afford pure **2** (100 mg, 61%) as a white solid that did not require further purification. ^{1}H NMR (400.16 MHz, DMSO-d^6, 298.15 K): δ_H 8.21+8.20 (doubled s, 1H, C=C$H^{triazol}$), 8.11 (d, *J*=8.7 Hz, 0.4H, H^{Ar}), 7.88 (d, *J*=8.7 Hz, 0.6H, H^{Ar}), 7.61 (d, *J*=2.5 Hz, 0.6H, H^{Ar}), 7.57 (d, *J*=9.0 Hz, 1H, N*H*Ac), 7.45-7.37 (2x dd, *J*=8.7 Hz, *J*=2.5 Hz, 1H, H^{Ar}), 7.31 (d, *J*=2.5 Hz, 0.4H, H^{Ar}), 4.82 (m, 2H, ArOCH_2CH$_2$N), 4.61 (m, 2H, ArOCH$_2$CH_2N), 4.52 (s, 2H, OCH_2C^{triazol}), 4.29 (d, *J*=8.5 Hz, 1H, H^1), 3.78 (m, 2H, 2H^{chain}), 3.70 (m, 1H, H^2), 3.64 (m, 1H, H^4), 3.58-3.48 (m, 11H, NCH_3 + $H^{6a,b}$ + 6H^{chain}), 3.42 (dd, *J*=10.6 Hz, *J*=3.3 Hz, 1H, H^3), 3.30 (t, *J*=6.1 Hz, 1H, H^5), 1.77 (s, 3H, NH*Ac*) ; ^{13}C NMR (100.62 MHz, DMSO-d^6, 298.15 K): δ_C 169.5, 161.1, 160.8, 144.0, 130.7, 128.7, 126.6, 124.4, 122.7, 121.8, 121.1, 108.5, 101.3, 75.3, 71.6, 69.7, 69.6, 68.9, 67.5, 66.7, 63.5, 60.5, 52.0, 48.8, 48.6, 22.9 ; HRMS (ES$^+$): *m/z* calculated for $C_{26}H_{37}N_6O_{11}$ [M+H]$^+$ 609.2520 found 609.2523

➢ *Electrochemical behaviour*

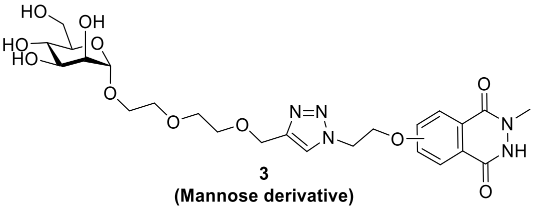

3
(Mannose derivative)

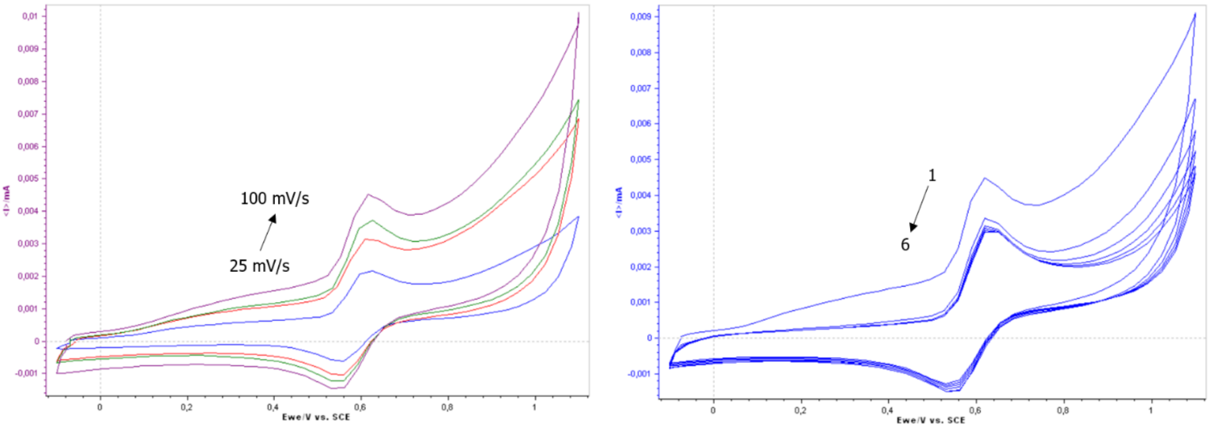

E_{ox} = 0.6 V *vs* SCE, reversible process

Supplementary Figure 6. **Electrochemical behaviour of Mannose derivative 3**
3 (1 mM) in MeCN/PB 50 mM pH 7.4 recorded at a 2 mm disc cathode: glassy carbon electrode, anode: platinum wire, reference: saturated calomel electrode. **(left)** Cyclic voltammetry at scan rate: 25, 50, 75 and 100 mV/s. **(right)** Multicyclic voltammetry (n= 6) at 100 mV/s

---

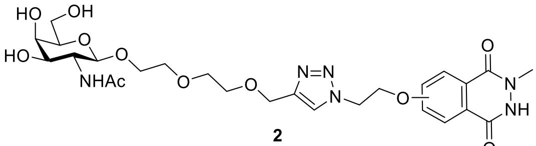

2
(GalNAc derivative)

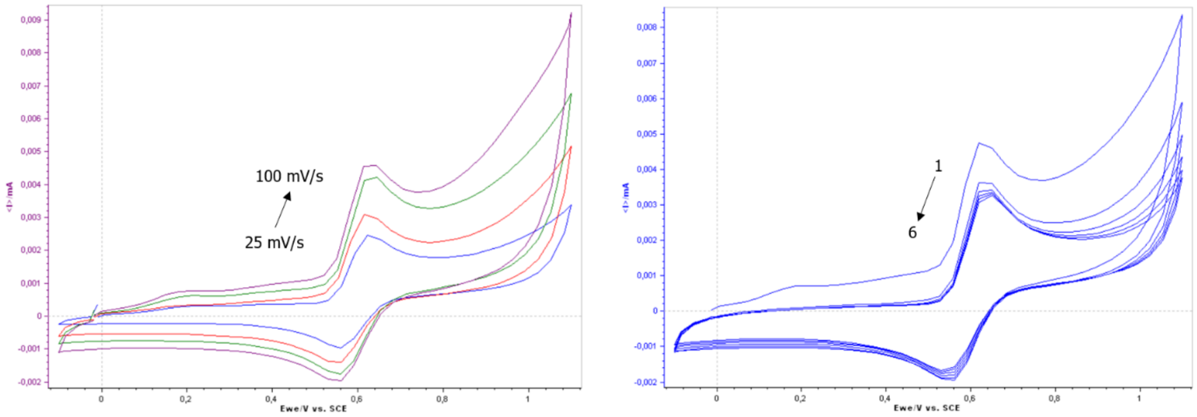

E_{ox} = 0.6 V *vs* SCE, reversible process

Supplementary Figure 7. **Electrochemical behaviour of GalNAc derivative 2**
2 (1 mM) in MeCN/PB 50 mM pH 7.4 recorded at a 2 mm disc cathode: glassy carbon electrode, anode: platinum wire, reference: saturated calomel electrode. **(left)** Cyclic voltammetry at scan rate: 25, 50, 75 and 100 mV/s. **(right)** Multicyclic voltammetry (n= 6) at 100 mV/s

3) Biotin derivative

➢ *Synthesis*

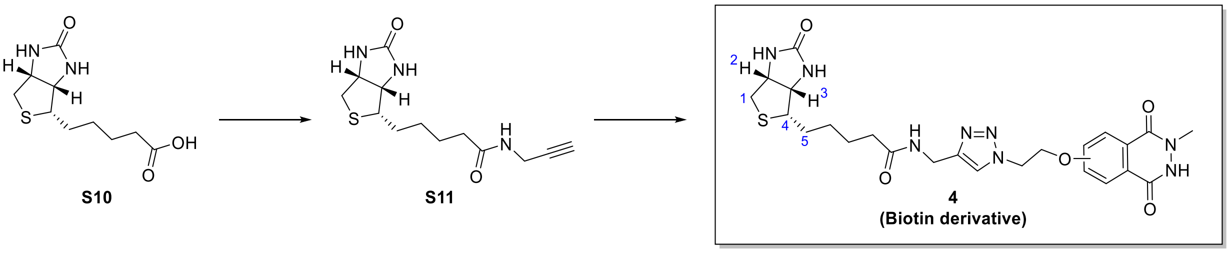

Supplementary Figure 8. **Synthesis of Biotin derivative 4**
Number 1-5 in **4** for ease of NMR attribution

Compound S11

Propargylamide derivative **S11** was prepared as described in literature from commercially available carboxylic acid **S10**.[2]

Compound 4 (Biotin derivative)

To a solution of **S11** (70 mg, 0.25 mmol, 1.1 equiv.) in dioxane (2 mL) were added azide derivative **1** (59 mg, 0.22 mmol, 1 equiv.) and sodium ascorbate (54 mg, 0.27 mmol, 1.2 equiv.). An aqueous solution (0.5 mL) of $CuSO_4.H_2O$ (24 mg, 0.13 mmol, 0.6 equiv.) was then added and reaction was heated up to 70 °C and stirred protected from light with aluminium. After 45 min, completion was monitored by TLC (Rf = 0.2 in 85:15 DCM/MeOH) and mixture was cooled to room temperature. Chelex resin® was added (2 spatula) and mixture was left stirred 10 min. Resin was filtered off and washed twice with MeOH. The obtained solution was concentrated under reduced pressure and purified by silica gel chromatography (90:10 to 85:15 DCM/MeOH) to afford **4** (61 mg, 50%) as a white solid. ^{1}H NMR (400.16 MHz, DMSO-d^6, 298.15 K): δ_H 8.25 (t, *J*=5.5 Hz, 1H, NH^{amide}), 8.11 (d, *J*=8.7 Hz, 0.3H, H^{Ar}), 8.00+7.99 (doubled s, 1H, C=C$H^{triazol}$), 7.88 (d, *J*=8.7 Hz, 0.7H, H^{Ar}), 7.56 (d, *J*=2.5 Hz, 0.7H, H^{Ar}), 7.44-7.35 (2x dd, *J*=8.7 Hz, *J*=2.5 Hz, 1H, H^{Ar}), 7.31 (d, *J*=2.5 Hz, 0.4H, H^{Ar}), 6.38 (br s, 1H, NH^{urea}), 6.33 (br s, 1H, NH^{urea}), 4.79 (m, 2H, N$CH_2CH_2O^{luminol}$), 4.58 (m, 2H, $NCH_2CH_2O^{luminol}$), 4.28 (d, *J*=5.5 Hz, 2H, N$CH_2C^{triazol}$, overlapped with m, 1H, H^2), 4.11 (m, 1H, H^3), 3.54+3.52 (doubled s, 3H, NCH_3), 3.08 (m, 1H, H^4), 2.80 (dd, *J*=12.3 Hz, *J*=5.1 Hz, 1H, H^{1a}), 2.57 (dd, *J*=12.3 Hz, *J*=2.0 Hz, 1H, H^{1b}), 2.09 (t, *J*=7.5 Hz, 2H, CH_2CONH^{amide}), 1.65-1.39 (m, 4H, H^{chain} (including H^{5a} + H^{5b})), 1.36-1.21 (m, 2H, H^{chain}) ; ^{13}C NMR (100.62 MHz, DMSO-d^6, 298.15 K): δ_C 172.9, 163.6, 162.0, 161.6, 146.1, 131.7, 129.6, 127.5, 124.2, 123.7, 122.7, 122.0, 109.5, 67.7, 61.9, 60.1, 56.3, 49.68, 35.9, 35.0, 29.1, 28.9, 26.1 ; HRMS (ES$^-$): *m/z* calculated for $C_{24}H_{29}N_8O_5S$ [M-H]$^-$ 541.1982 found 541.1984

➢ *Electrochemical behaviour*

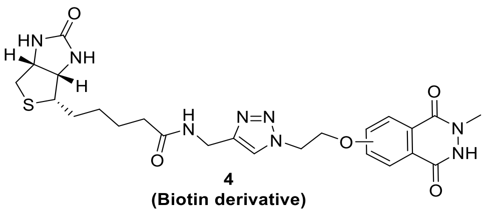

4
(Biotin derivative)

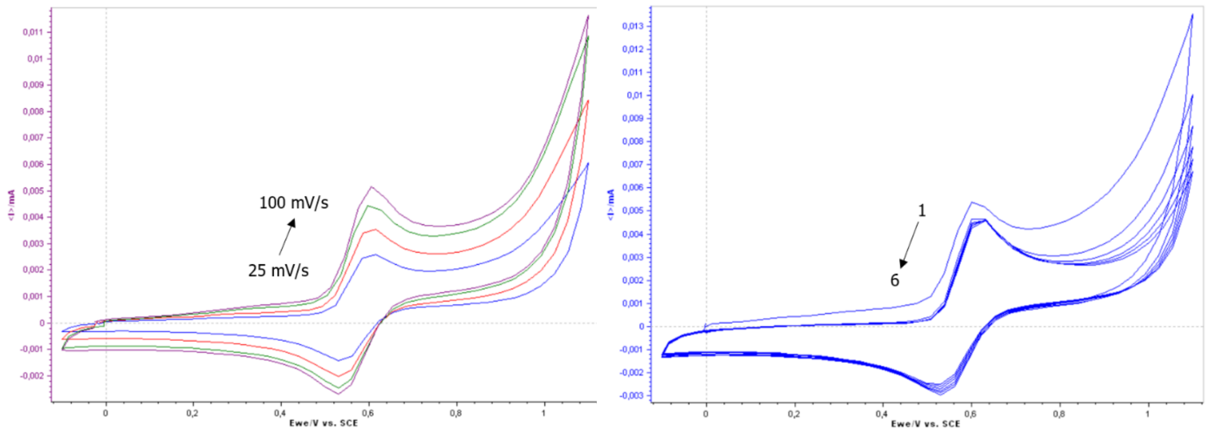

E_{ox} = 0.6 V *vs* SCE, reversible process

Supplementary Figure 9. **Electrochemical behaviour of Biotin derivative 4**
4 (1 mM) in MeCN/PB 50 mM pH 7.4 recorded at a 2 mm disc cathode: glassy carbon electrode, anode: platinum wire, reference: saturated calomel electrode. **(left)** Cyclic voltammetry at scan rate: 25, 50, 75 and 100 mV/s. **(right)** Multicyclic voltammetry (n= 6) at 100 mV/s

II. eY-click on AAV2: Procedures and characterizations

Electrobioconjugation procedure (*1 mL scale setup*)

In a 2 mL low-binding vial, 100 µL of AAV2-GFP ($1^{E}12$ vg in dPBS pH 7.4, produced by the vector production center-CPV of Nantes UMR1089 gene therapy laboratory) were added to 900 µL dPBS pH 7.4 containing the appropriate quantity of carbohydrate luminol derivative **2** or **3** (final conc. 0.5 mM or 5 mM). The 1 mL scale setup was assembled (**Figure S3**) and 750 mV vs Ag/AgCl were applied during the studied time at room temperature under gentle orbital shaking. After modification, the excess of unreacted luminol anchor was removed by dialysis in four successive rounds against dPBS pH 7.4 (+0.001% Poloxamer) in a 10 kDa MWCO cassette.

Characterizations

Titration of viral genomes (vg)

To determine the titer (vg/mL) of all AAV samples, 3 µL were treated with 20 units of DNase I (Roche #04716728001) at 37 °C for 45 min to remove residual DNA in vector samples. After treatment with DNase I, 20 µL of proteinase K (20 mg/mL, from MACHEREY-NAGEL®) was added and the mixture was incubated at 70 °C for 20 min. An extraction column (NucleoSpin®RNA Virus) was then used to extract DNA from purified AAV vectors. Quantitative real time PCR (qPCR) was performed with a StepOnePlus™ Real-Time PCR System Upgrade (Life Technologies). All PCRs were performed with a final volume of 20 µL, including primers and probes targeting the ITR2 sequence, PCR Master Mix (TaKaRa), and 5 µL of template DNA (plasmid standard or sample DNA).[3] qPCR was carried out with an initial denaturation step at 95 °C for 20 seconds, followed by 45 cycles of denaturation at 95 °C for 1 second and annealing/extension at 56 °C for 20 seconds. Plasmid standards were generated with seven serial dilutions (containing 108 to 102 plasmid copies).

Dot Blot, Western Blot and Silver Staining

For dot blot analysis, nitrocellulose membrane was soaked briefly in PBS prior to assembling the dot blot manifold (BioRad), then AAV vectors (2.10^{10} vg) were loaded. The obtained nitrocellulose membrane was then treated for the appropriate characterization (see below: capsid integrity or carbohydrate detection). For silver nitrate or western blot, all AAV vectors (2.10^{10} vg) were denatured at 100 °C for 5 min using Laemmli sample buffer (5 µL) and separated by SDS-PAGE on 10% Tris-glycine polyacrylamide gels (Life Technologies). Precision Plus Protein All Blue Standards (BioRad) were used as a molecular-weight size marker. After electrophoresis at 120 V during 200 min, gels were either silver stained (PlusOne Silver Staining Kit, Protein from GE Healthcare®) or transferred onto nitrocellulose membranes for Western blot. A 25 mM Tris/192 mM glycine/0.1 (w/v) SDS/20% MeOH buffer was used to transfer proteins during 10 min at 150 mA in a Trans-Blot SD Semi-Dry Transfer Cell (from BioRad®). The obtained nitrocellulose membrane was then treated for the appropriate characterization (See below: viral capsid proteins or carbohydrate detection).

Capsid integrity: Membrane was saturated for 2 h at RT with PBS containing 5% semi-skimmed milk and 0.1% tween. After saturation, the membrane was probed with primary antibody to mouse anti-capsid

A20 (from Kleinschmidt®, diluted in milk solution 1:20) overnight at 4 °C. Then, membrane was washed thrice 15 min at RT with PBS-0.1%Tween, and probed with secondary antibody anti-mouse-HRP (from Dako®, diluted in milk solution 1:2000) during 1h30 at RT. Membrane was finally washed thrice 15 min at RT with PBS-0.1%Tween and detection of bands was performed by local treatment with H_2O_2/luminol during 1 min followed by chemiluminescence visualization on X-ray films.

Viral capsid proteins detection: Membrane was saturated for 2 h at RT with PBS containing 5% semi-skimmed milk and 0.1% tween. After saturation, the membrane was probed with primary antibody to rabbit polyclonal anti-AAV capsid proteins (from PROGEN Biotechnik®, diluted in milk solution 1:2000) overnight at 4 °C. Then, membrane was washed thrice 15 min at RT with PBS-0.1%Tween, and probed with secondary antibody anti-rabbit-HRP (from Jackson®, diluted in milk solution 1:20000) during 1h30 at RT. Membrane was finally washed thrice 15 min at RT with PBS-0.1%Tween and detection of bands was performed by local treatment with H_2O_2/luminol during 1 min followed by chemiluminescence visualization on X-ray films.

Carbohydrate detection: Membrane was saturated for 2 h at RT with PBS containing 1% gelatin, 0.1% igepal and 0.1% tween. After saturation, the membrane was probed with Soybean Agglutinin-Fluorescein lectin (from Vector Laboratories®, diluted in PBS-0.1%Tween solution 1:200) for GalNAc detection or Concanavalin A-Fluorescein lectin (from Vector Laboratories®, diluted in PBS-0.1%Tween solution 1:200) for mannose detection, overnight at 4 °C. Then, membrane was washed thrice 15 min at RT with PBS-0.1%Tween, and probed with secondary antibody anti-Fluorescein-HRP (from abcam®, diluted in PBS-0.1%Tween solution 1:5000) during 1h30 at RT. Membrane was finally washed thrice 15 min at RT with PBS-0.1%Tween and detection of bands was performed by local treatment with H_2O_2/luminol during 1 min followed by chemiluminescence visualization on X-ray films.

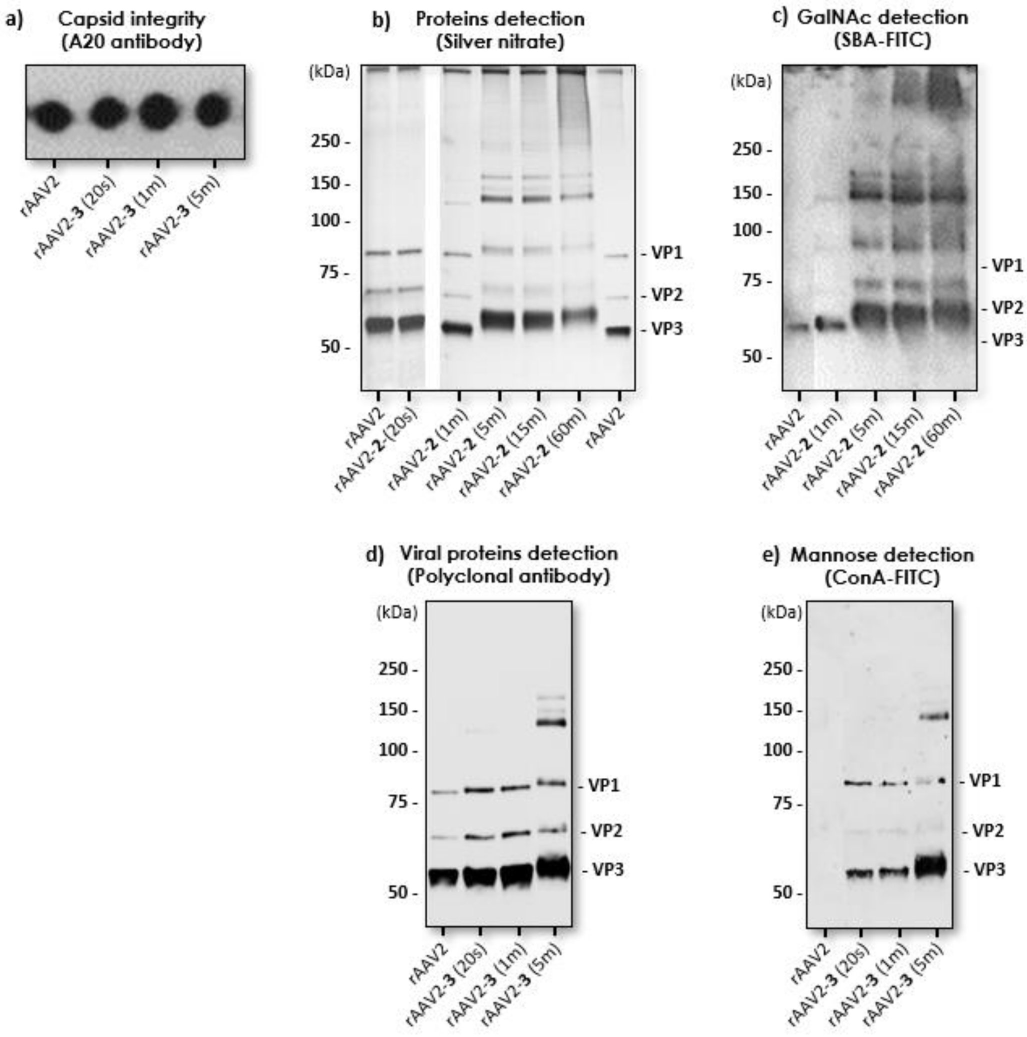

Supplementary Figure S10. **Proteins and sugars detection on viral capsids rAAV2**
a) Dot blot analysis of viral vectors (2.10^{10} vg) to detect the assembled capsid using the A20 antibody. **b)** Silver nitrate staining of viral vectors (2.10^{10} vg) after denaturation to detect all proteins, n=1. **c)** Western blot analysis of viral vectors (2.10^{10} vg) after denaturation to detect surface-conjugated GalNAc using a labeled soybean agglutinin (GalNAc-binding lectin), n=1. **d)** Western blot analysis of viral vectors (2.10^{10} vg) after denaturation to detect the three constitutive viral proteins (VP1/VP2/VP3 1:1:10) using a polyclonal anti-VPs antibody, n=1. **e)** Western blot analysis of viral vectors (2.10^{10} vg) after denaturation to detect surface-conjugated Man using a labeled ConA (Man-binding lectin), n=1. Source data are provided as a source data file.

In vitro transduction

The infectivity of each sample was measured as follows. HUH7 or HEK293 cells were seeded in DMEM with 10% FBS serum and 1% penicillin-streptomycin in 6-well culture plates at a density of 10^6 cells/well. Cells were then incubated overnight at 37 °C with 5% CO_2 to reach 50% confluence. Then, AAV samples were prepared by serial dilution considering the studied *m*ultiplicity *o*f *i*nfection (MOI = n virus / n cells, varying from 10^3 to 10^4) and 2 μL of the samples were added to separate wells in the 6-well plates. The latter were incubated at 37 °C for 24 h. AAV-GFP-infected cells were detected and quantified by fluorescence microscopy and flow cytometry on a BD-LSRII Flow Cytometer (BD Bioscience). All data

were processed by FlowJo (V10, Flowjo LLC, Ashland, OR). Data are shown as mean ± SD (standard deviation). Distinct samples were measured (n=3).

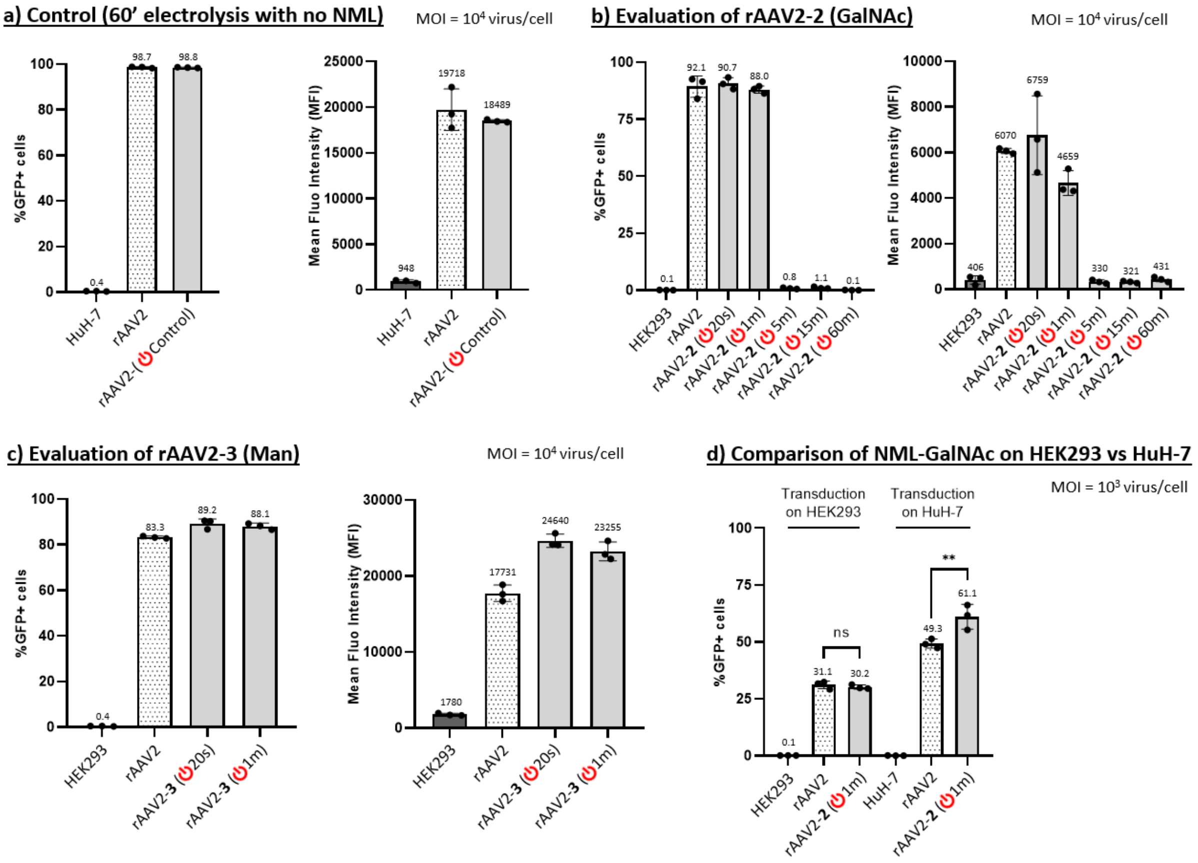

Supplementary Figure 11. **Transduction evaluation of viral vectors (carrying a GFP reporter gene) on HuH-7 or HEK293 cells after 48 h incubation.**

a) Evaluation of the control experiment where the current was applied to a solution of rAAV2 and a GalNAc derivative with no NML moiety during 1 h. **b)** Evaluation of all rAAV2-GalNAc electro-functionalized vectors. **c)** Evaluation of all rAAV2-Man electro-functionalized vectors. **d)** comparison of transduction efficiency of rAAV2-**2**-1min on HEK293 (without GalNAc receptors) and HuH-7 (with GalNAc receptors). Statistical significance was assessed using one-way ANOVA tests with Dunnett's multiple comparisons (means comparison) and is presented as ns (not significant), $^{**}P \leq 0.002$. For all the cases described here, %GFP+ cells and Mean Fluorescence Intensity (MFI) were obtained by flow cytometry. MOI are given top right. n=3 biologically independent samples. Data are presented as mean values +/- SD. Source data are provided as a source data file.

III. eY-click on bacterial strains: Procedures and characterizations

Electrobioconjugation procedure (*5 mL scale setup*)

TOP10 *Escherichia coli* or *Staphylococcus epidermidis* strains were precultured in lysogeny broth (LB) until optical density (OD) 0.4 approx. Then, 250 μL of preculture media were diluted in 25 mL LB and incubated at 450 g and 20 °C during 16 h (OD = 0.4 approx.). Bacteria were centrifuged at 20000 g during 5 min, supernatant was withdrawn followed by resuspension in 25 mL PBS pH 7.4. Next, to 2.5 mL of the bacterial strain (*E. Coli* or *Staph. Epidermidis*, optical density = 0.4) solution in PBS pH 7.4 were added 2.5 mL of 2 mM azide luminol derivative **1** (final conc. 1 mM) solution in PBS pH 7.4. The 5 mL scale setup was assembled and 750 mV vs Ag/AgCl were applied during the studied time at room temperature at 500 rpm. After modification, the excess of unreacted luminol anchor was removed by performing three times the following sequence: *i)* centrifugation (20000 g during 1 min), *ii)* supernatant withdrawal, *iii)* bacteria resuspension in 1 mL PBS. At the end of 3rd sequence, bacteria were resuspended in 190 μL PBS pH 7.4 and 10 μL of a 2 mM DBCO-PEG$_4$-CR110 **5a** (obtained from Jena Bioscience®) solution (final conc. 0.1 mM) in DMSO were added. The sample was incubated at 23 °C in the dark during 1 h under moderate orbital shaking. Then, the excess of unreacted cyclooctyne was removed by performing four times the previous centrifugation/removal/washings (resuspension included 0.5% DMSO for the two first sequences). At the end of 4th sequence, bacteria were resuspended in the appropriate volumes/solutions for characterizations.

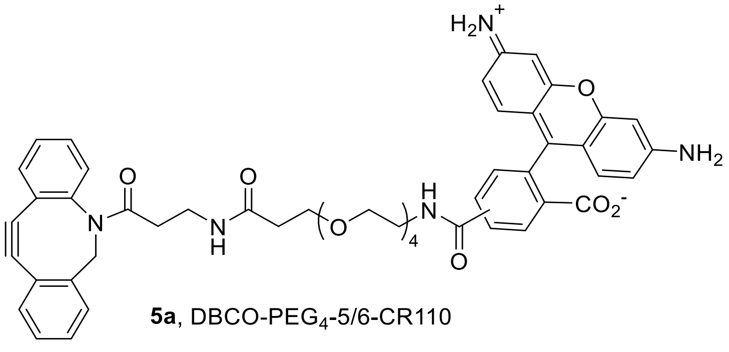

5a, DBCO-PEG$_4$-5/6-CR110

Characterizations

Bacteria viability

Bacteria viability was evaluated by their ability to grow in culture conditions. The longest electro-conjugation conditions (1 h) was performed in triplicate and evaluated. Directly after the electro-conjugation step, 300 μL of the samples (OD_{600}=0.6) were taken and treated 2 times with the washing sequence: centrifugation (20000 g during 2 min) - supernatant withdrawal – resuspension in 1 mL LB (final resuspension in 300 μL LB). 80 μL of the washed samples were seeded (each condition in triplicate)

in a 24-well plate, diluted to 1 mL LB (OD_{600}=0.05), and incubated at 450 g at 20 °C. OD_{600} were regularly measured on an Infinite M1000 Microplate reader from TECAN using Magellan Software.

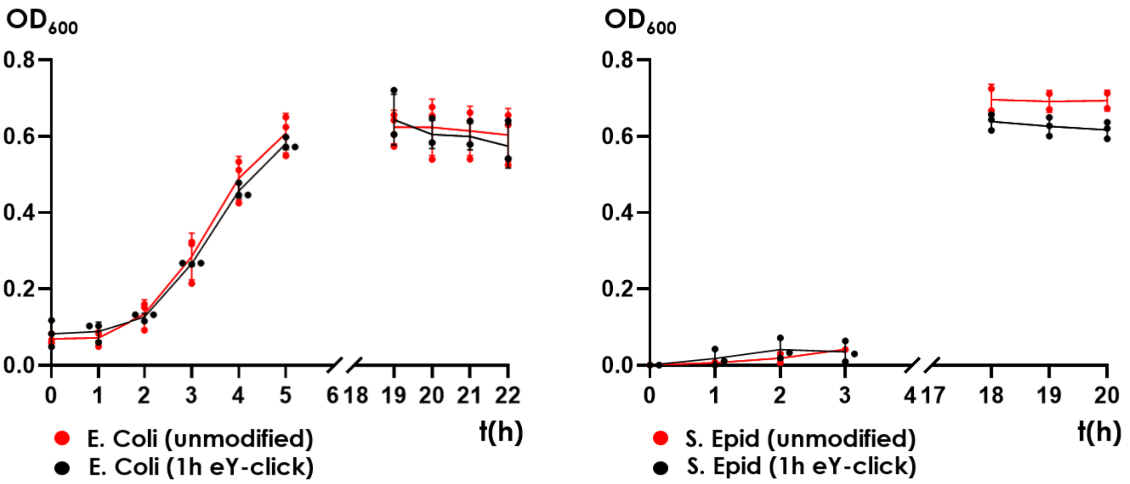

Supplementary Figure 12. **Bacterial growth curves of 1-labeled bacteria samples after 1h eY-click compared to untreated strains.**

Each sample was cultured (*n*=3) and OD_{600} was regularly measured. Source data are provided as a source data file

Fluorescence microscopy and flow cytometry

Electrobioconjugation efficiency was visualized by fluorescence microscopy (FITC excitation conditions) using Nikon Eclipse NI-E microscope (data treated with NIS software) and quantified by flow cytometry using CYTOFLEX cytometer from Beckman Coulter® – Life Sciences (data treated with FlowJo software).

***E. Coli* experiments**

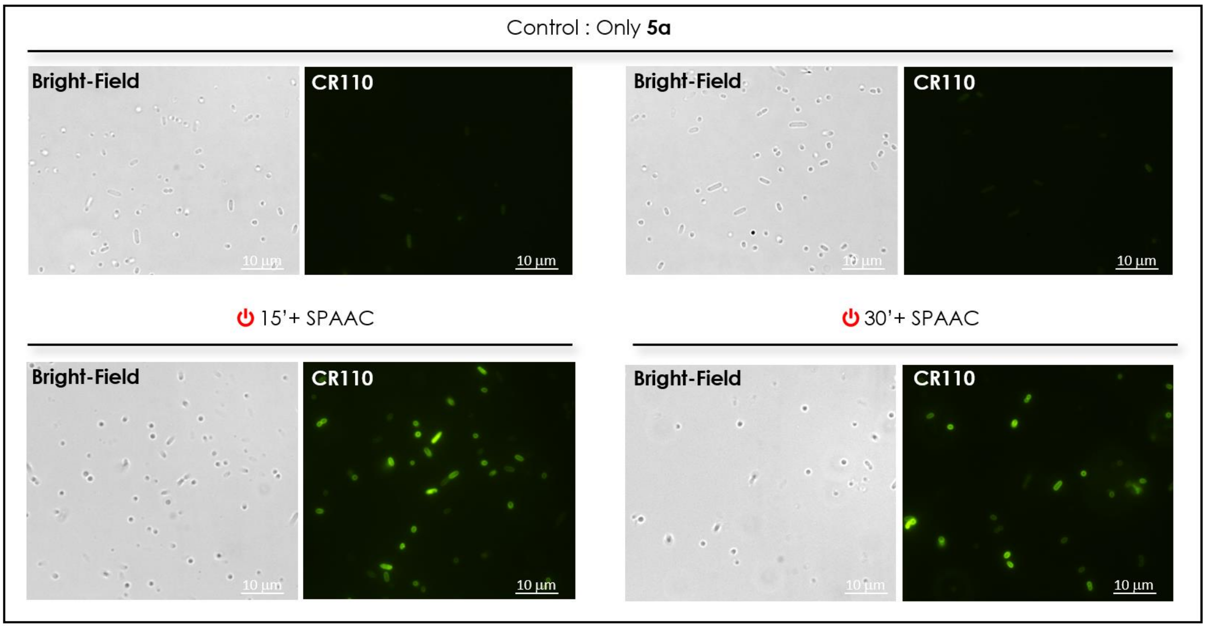

S. Epidermidis experiments

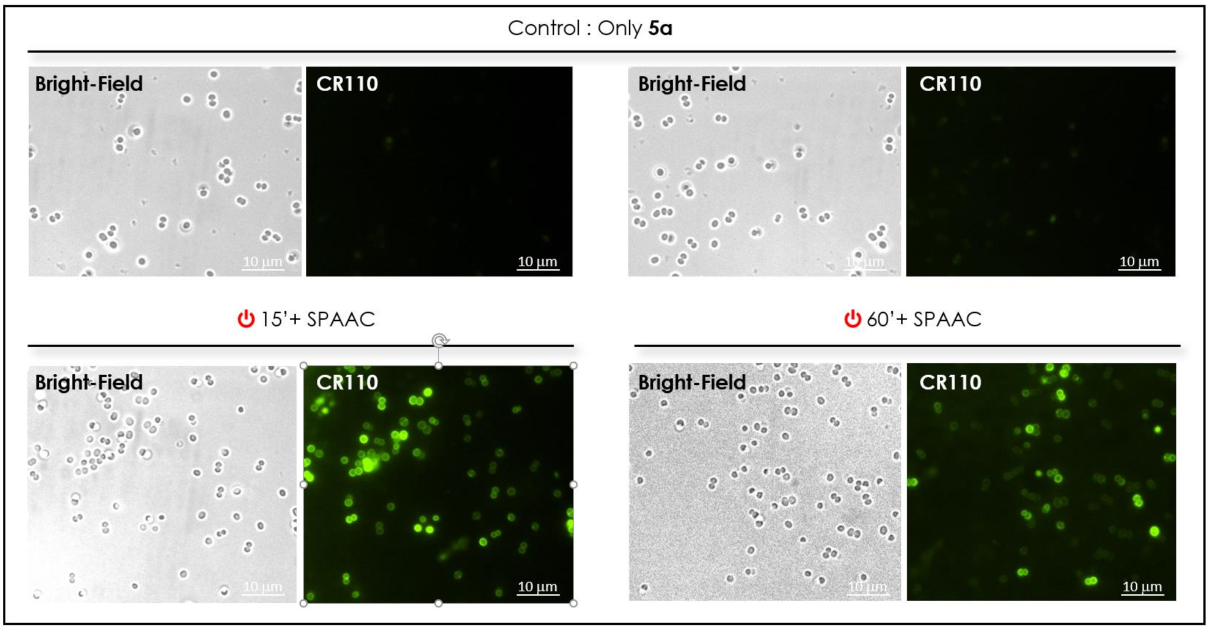

Supplementary Figure 13. Optical and fluorescence images of *E. coli* and *S. epidermis* samples subjected to only 5a (control) or eY-click and SPAAC. All experiments were performed in duplicate with same results.

Proteins membrane extraction and SDS-PAGE analysis

Materials and methods: 800 µL of each sample were split in 2x400 µL. The latter were centrifuged and supernatants removed. First part was resuspended in 50 µL of resuspension buffer (sodium phosphate buffer 50 mM pH 7.4/300 mM NaCl/2 mM $MgCl_2$/DNASE 1000X/Lysozyme 100X/Protease inhibitor cocktail 200X) and 10 µL of Laemmli 6X buffer were added (Total fraction). Second part was resuspended in 500 µL of resuspension buffer and lysed by 3x periodic 5sec ON/OFF ultrasonication followed by centrifugation at 12000g during 30 min. Supernatant was taken off and concentrated using 3K MWCO VWR® centrifugal filters until 80 µL final volume, and 10 µL of Laemmli 6X buffer were added (Cytosolic fraction). The remaining centrifugated pellet was resuspended in 50 µL of 8M urea/50 mM NaH_2PO_4/300 mM NaCl buffer and 10 µL of Laemmli 6X buffer were added (Membrane fraction). For each sample, the 3 fractions were heated at 95 °C during 5 min (10 min for total fraction) and 10 µL (20 µL for cytosolic fractions) were deposed on a 12 % acrylamide gel. Proteins were separated at 90 V during 10 min then at 150 V during 1h15. Gels were visualized under UV, washed 3x 10 min with water and then stained with Coomassie brilliant blue: coloration overnight and 3x 30 min discoloration with water.

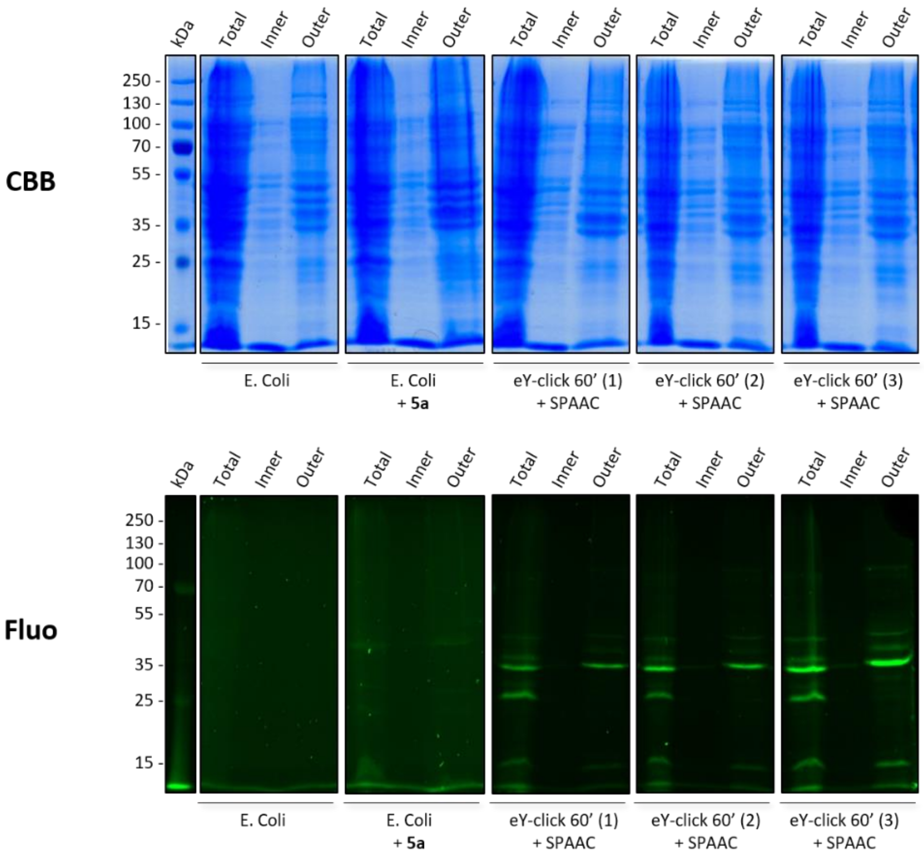

Supplementary Figure 14. Labelled proteins from *E. coli*.

SDS-PAGE analysis on gels of total, inner and outer proteins fractions of untreated bacteria, bacteria treated only with **5a**, and bacteria treated by 1h eY-click with **1** in three different electro-bioconjugation experiments. Visualization by CBB and fluorescence. Source data are provided as a source data file.

Gel quantification

Image Name	Channel	Name	Signal	Total	Area	Bkgnd.
0004985_06	700	00001	91405,84082	113530,4531	8360	2,64648438
0004985_06	700	00002	33530,81641	68881,20313	8360	4,22851563
0004985_06	700	00003	34060,27246	51074,17969	8360	2,03515625
0004985_06	700	00004	-817,6230469	16710,61914	8360	2,09667969
0004985_06	700	00005	86680,02539	106371,7422	8360	2,35546875
0004985_06	700	00006	41069,42773	68190,44531	8360	3,24414063
0004985_06	700	00007	31585,73438	56820,85156	8360	3,01855469

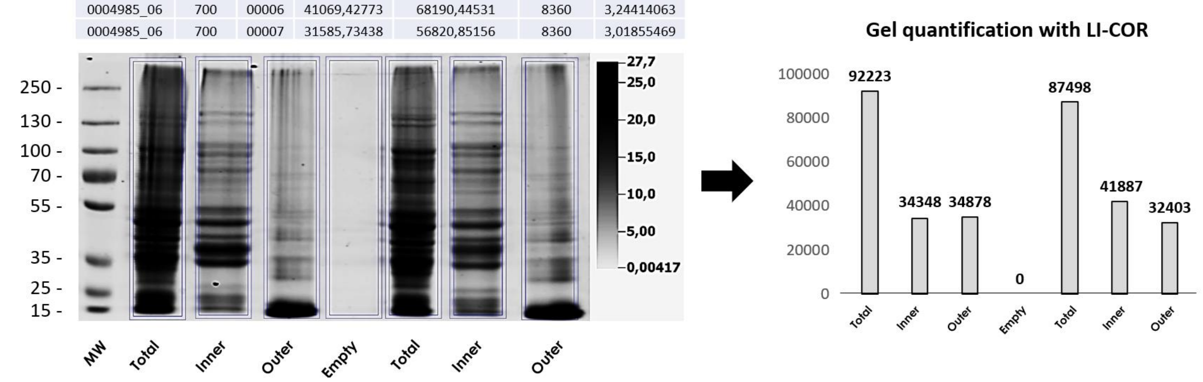

Supplementary Figure 15. **Quantification of proteins on gel.**

Quantification of proteins migrated in lanes "total", "inner" and "outer" after the fractionation protocol and electrophoresis. Quantification using a LI-COR imaging system. Experiments were performed in duplicate. Source data are provided as a source data file

IV. eY-click on living cells: Procedures and characterizations

Electrobioconjugation procedure with azido derivative (*1 mL scale setup*)

Cells (HEK293 or HeLa) were cultured with 10% FBS serum and 1% penicillin-streptomycin at 37 °C with 5% CO_2. The cells were trypsinized and harvested in PBS pH 7.4 at a concentration of 6.10^6 cells/mL. Then, in a 2 mL low-binding vial, 500 µL of the cell solution (final conc. 3.10^6 cells/mL) were added to 500 µL of a 2 mM solution of **1** in PBS pH 7.4 (final conc. 1 mM). The 1 mL scale setup was assembled (Figure S3) and 750 mV vs Ag/AgCl were applied during the studied time at room temperature under gentle orbital shaking. After modification, the excess of unreacted luminol anchor was removed by performing three times the following sequence: *i)* centrifugation (1200 g during 2 min), *ii)* supernatant withdrawal, *iii)* cells resuspension in 1 mL PBS. At the end of 3rd sequence, cells were resuspended in 250 µL PBS pH 7.4 and 250 µL of a 0.2 mM DBCO-PEG$_4$-FAM **5b** (from Jena Bioscience®) solution in PBS were added (final conc. 0.1 mM). The sample was incubated at 23 °C in the dark during 1 h under moderate orbital shaking. Then, the excess of unreacted cyclooctyne was removed by performing three times the previous centrifugation/removal/washings with 1 mL PBS. At the end of 3rd sequence, modified cells were resuspended in the appropriate volumes for characterizations.

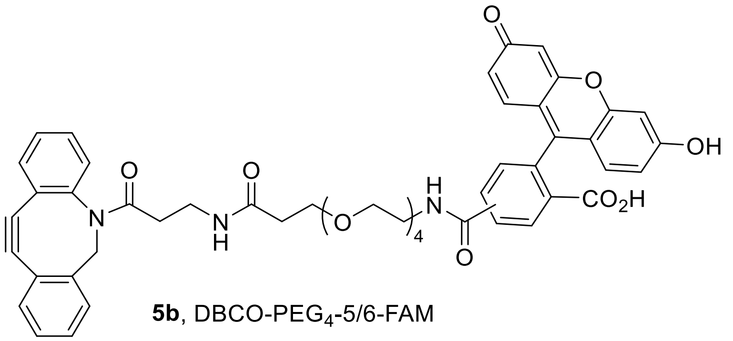

5b, DBCO-PEG$_4$-5/6-FAM

Electrobioconjugation procedure with biotin derivative (*1 mL scale setup*)

HeLa cells were cultured with 10% FBS serum and 1% penicillin-streptomycin at 37 °C with 5% CO_2. The cells were trypsinized and harvested in PBS pH 7.4 at a concentration of 6.10^6 cells/mL. Then, in a 2 mL low-binding vial, 500 µL of the cell solution (final conc. 3.10^6 cells/mL) were added, centrifuged to remove supernatant and 1 mL of 1 mM solution of **4** in PBS pH 7.4 (final conc. 1 mM) was added (**4** has poor solubility in aqueous media and it was helped with ultrasonic bath). The 1 mL scale setup was assembled (**Figure S2**) and 750 mV vs Ag/AgCl were applied during the studied time at room temperature under gentle orbital shaking. After modification, the excess of unreacted luminol anchor was removed by performing three times the following sequence: *i)* centrifugation (1200g during 2 min), *ii)* supernatant withdrawal, *iii)* cells resuspension in 1 mL PBS. At the end of 3rd sequence, cells were resuspended in 50 µL of a Streptavidin-FAM solution (obtained from ThermoFisher®) and incubated at 37 °C during 1h under moderate orbital shaking. Then, non-complexed Streptavidin was removed by performing three times the previous centrifugation/removal/washings with 1 mL PBS. At the end of 3rd sequence, modified cells were resuspended in the appropriate volumes for characterizations.

Characterizations

Cell viability

Cell culture: Cell viability after modification was evaluated by cell culture. Unmodified, controls and conjugated cells were seeded in DMEM with 10% FBS serum and 1% penicillin-streptomycin in a 24-well culture plate and incubated at 37 °C with 5% CO_2. Growth ability and confluences were evaluated, quantified and compared by microscopy and using Vi-CELL XR after 24 h, 48 h and 72 h.

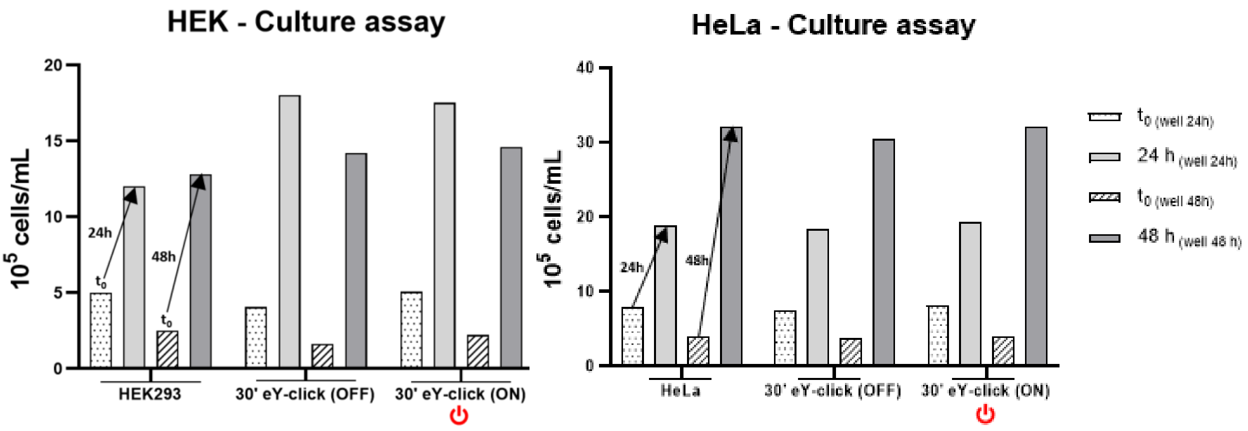

Supplementary Figure 16. Cell growth ability after eY-click

Cells quantification by Vi-CELL XR after 24h or 48h culture of samples. The ability to multiply was conserved for all samples. Source data are provided as a source data file.

Viability test with trypan blue: Approx. 5.10^5 cells in 600 μL PBS were subjected to automatized trypan blue viability test using Vi-CELL XR (from Beckman Coulter, Life Sciences). % of viable cells is calculated from the ratio of viable cells and total cells. Viability was also evaluated after 24h and 48h culture of electro-labelled cells.

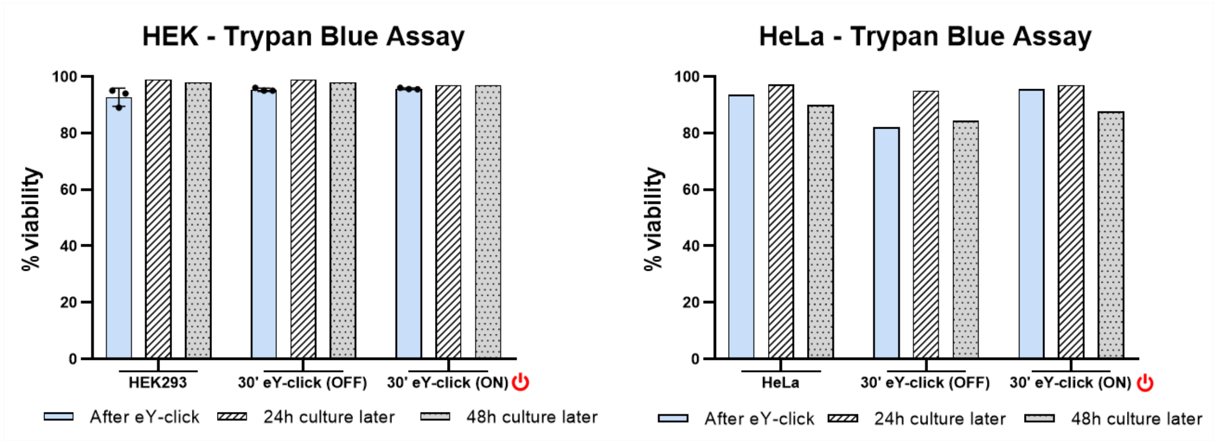

Supplementary Figure 17. Cell viability after eY-click

Automatized Trypan Blue viability test (Vi-CELL XR). All samples were found viable. Data are presented as mean values +/- SD of three independent electro-conjugation experiments for viability tests right after eY-click and as the obtained value (n=1) after 24h and 48h cell culture. Source data are provided as a source data file.

Viability test with Zombie Yellow and Annexin V: Approx. 2.10^6 electro-conjugated cells were resuspended in 1 mL of freshly prepared Zombie Yellow staining buffer (100 µL Zombie Yellow BV605 from Biolegend® diluted in 1 mL with Brilliant Violet Stain Buffer from BD Biosciences®) and incubated at RT in the dark. Centrifugation/supernatant withdrawal/PBS washing sequence was performed twice and the cells were resuspended in 30 µL of Annexin V 1X buffer and 1.5 µL of PE-CF594 Annexin V (from Fischer Scientific®) were added. After 15 min incubation at RT in the dark, samples were diluted with 400 µL of Annexin V 1X buffer and analyzed by flow cytometry to evaluate proportions of dead cells and apoptotic cells.

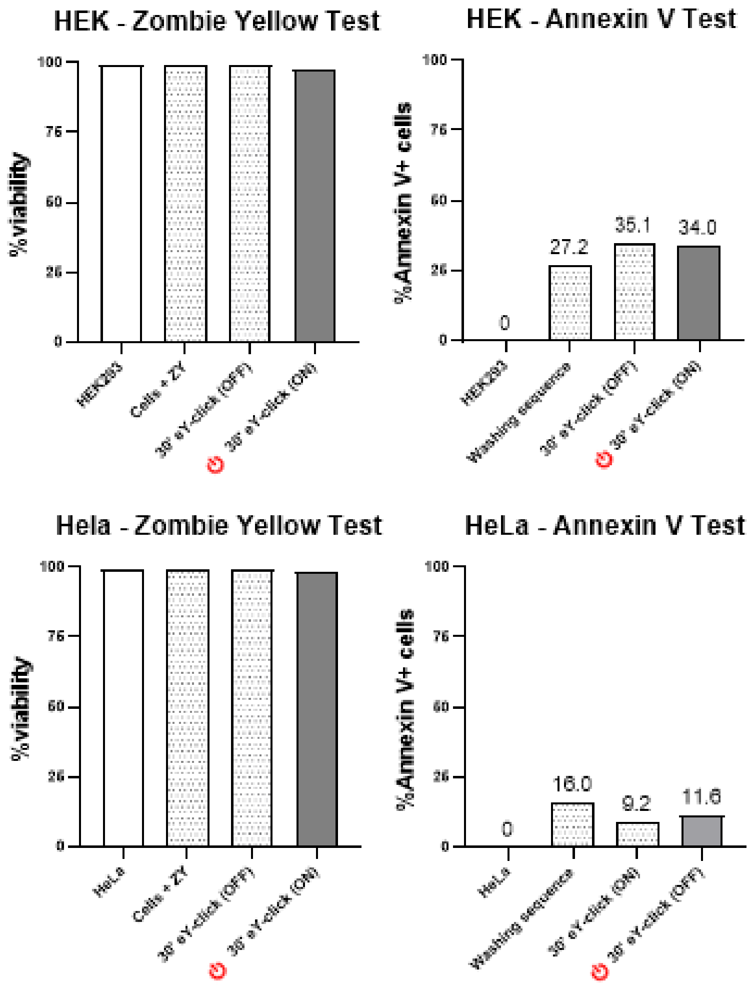

Supplementary Figure 18. Zombie Yellow and Annexin V viability tests.
Zombie Yellow test led to fully conserved viability. For Annexin V assay, untreated cells labelled with annexin V were used as reference. The electro-bioconjugation protocol didn't compromise cells viability, as an overall comparison with experiments without electrical current (eY-click OFF) or without NML derivative (Washing sequence). HEK cell line seems more stressed by the protocol, probably because these are adherent cells. Source data are provided as a source data file.

Flow cytometry

Electrobioconjugation efficiency was evaluated and quantified by flow cytometry. At the end of the electro-conjugation procedure, samples were directly analysed on BD-LSRII Flow Cytometer (BD Bioscience) considering FITC functionalization and detection. All data were processed by FlowJo (V10, Flowjo LLC, Ashland, OR). Data are shown as mean ± SD (standard deviation). Independent replicates were measured (n=3). Statistical significance was assessed using one-way ANOVA tests with Dunnett's multiple comparisons (means comparison) and is presented as ***P ≤ 0.0001.

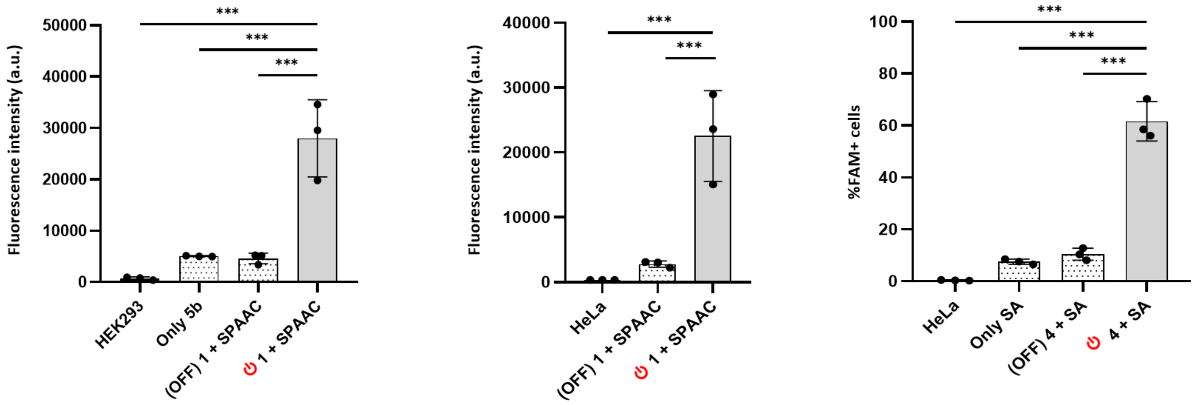

Supplementary Figure 19. eY-click efficiency on HEK293 and HeLa

Results obtained from flow cytometry (FACS) for (left to right) HEK293 eY-click with **1** then SPAAC with **5b**, HeLa eY-click with **1** then SPAAC with **5b**, HeLa eY-click with **4** then probing with SA-FITC. *n*=3 biologically independent samples. Data are shown as mean ± SD (standard deviation). Independent replicates were measured (n=3). Statistical significance was assessed using one-way ANOVA tests with Dunnett's multiple comparisons (means comparison) and is presented as ***P ≤ 0.0001. Source data are provided as a source data file.

Fluorescence confocal microscopy

Cells treatment: Membrane mapping was performed by incubating samples with 100 µL of Wheat Germ Agglutinin-AF647 lectin solution (from Invitrogen™, diluted with PBS 1:1000) 30 minutes at 4 °C. Two centrifugation/supernatant withdrawal/washings were performed with 250 µL of Perm/Wash Buffer (BD Cytofix/Cytoperm™ Fixation/Permeabilization Solution Kit, Fisher Scientific). The cells were then permeabilized with 100 µL of Fixation/Permeabilization solution (BD Cytofix/Cytoperm™ Fixation/Permeabilization Solution Kit, Fisher Scientific) and incubated 20 minutes at 4 °C. Two washings were performed the same way as before and nuclei were stained with 100 µL of DAPI solution (from Sigma Aldrich®, diluted with PBS 1:1000) during 15 minutes at RT.

Confocal Microscopy: Fluorescence imaging of the cells was performed on a Nikon A1R confocal microscope using a 60×/1.4 objective. The different channels were recorded as follows: excitation 405 nm: emission recorded from 425 to 475 nm; excitation 488 nm: emission recorded from 500 to 550 nm; and excitation 640 nm, emission recorded from 660 to 740 nm. The gain, offset and the power of lasers

were adjusted as needed. Three-dimensional digital images were collected using NIS-Elements confocal software and appropriate fluorescence filters. We acknowledge the IBISA MicroPICell facility (Biogenouest), member of the national infrastructure France-Bioimaging.

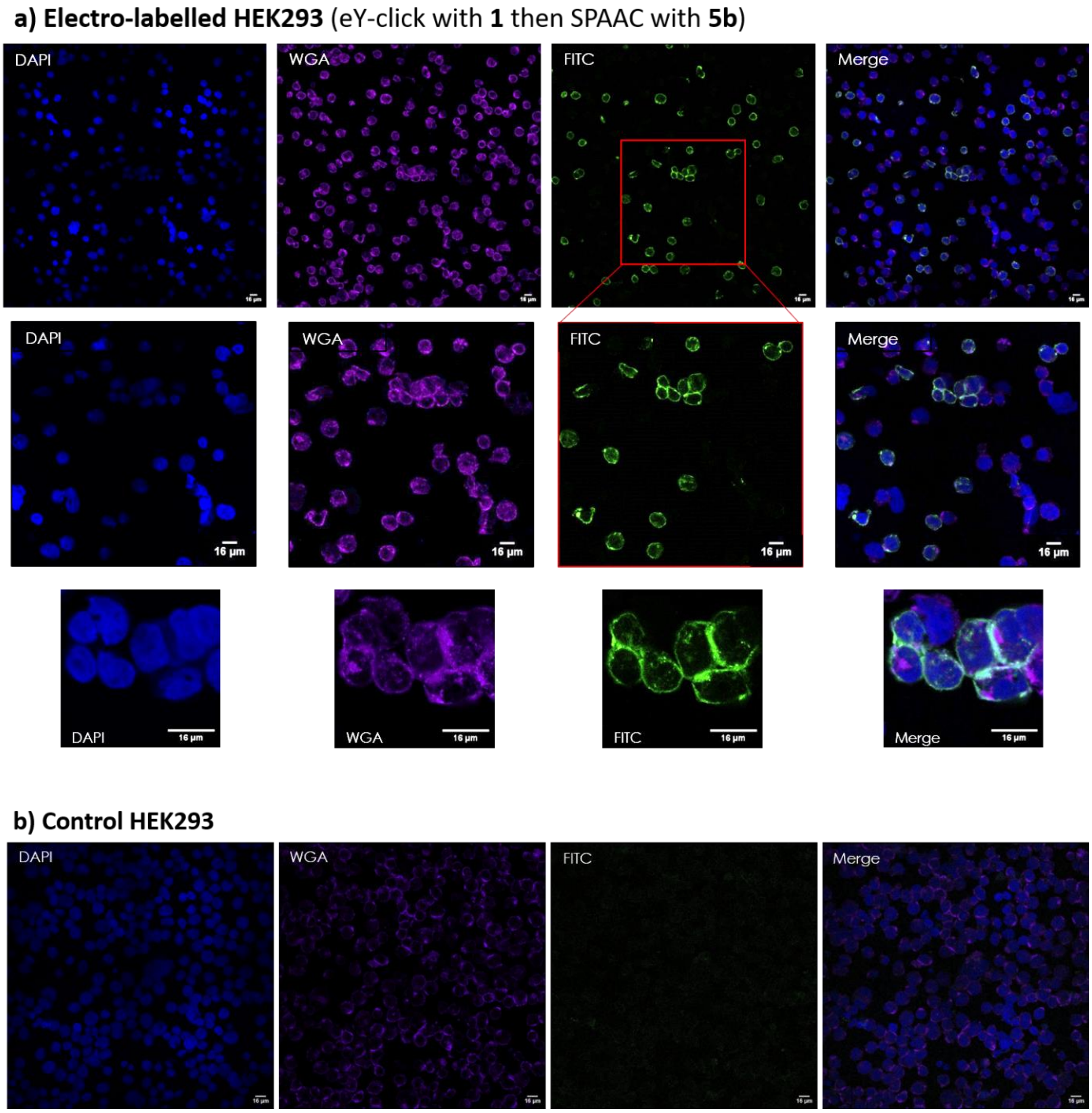

Supplementary Figure 20. Confocal microscopy images of HEK293 cells

a) treated by 30 min eY-click with **1** and 1 h SPAAC with **5b**, and **b)** untreated. WGA stands for membrane staining, DAPI is nuclei staining, FITC highlights fluorescent probe **5b**. Merge image showcases co-localization of WGA and FITC for eY-click experiment (n=1). Scale are given bottom right.

V. NMR Spectra

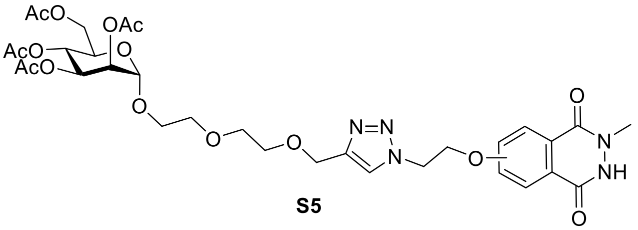

S5

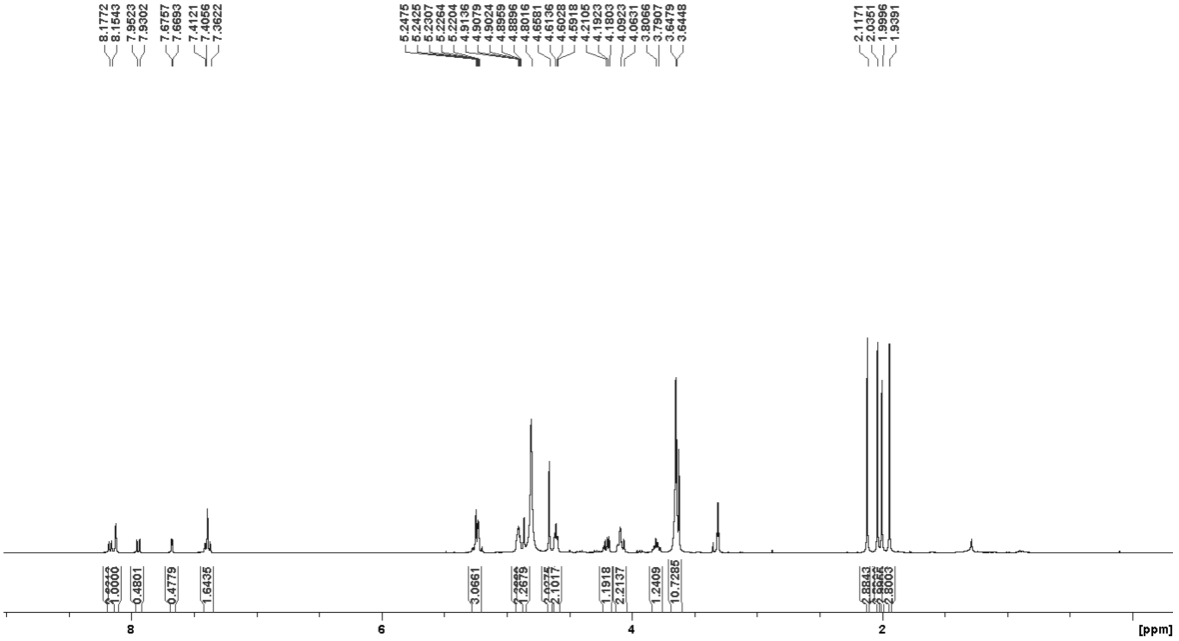

Supplementary Figure 21. ^{1}H NMR spectra of compound S5

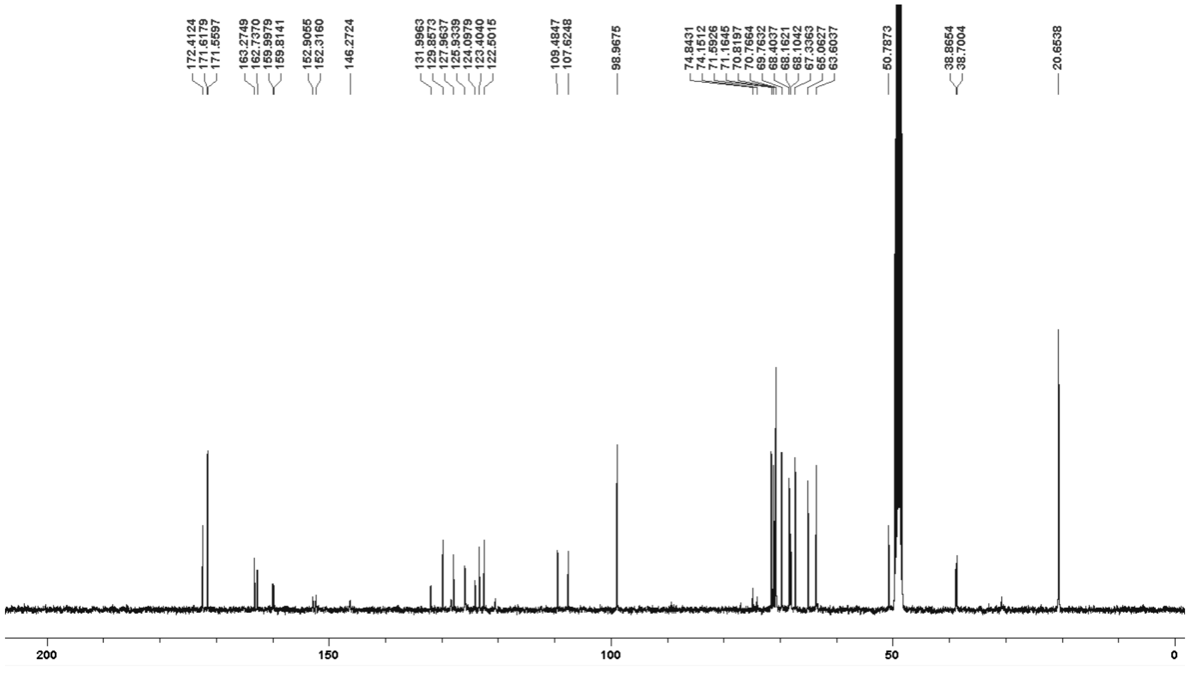

Supplementary Figure 22. ^{13}C NMR spectra of compound S5

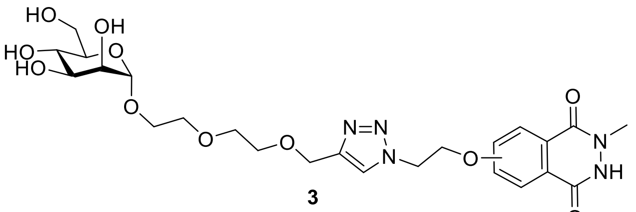

3
(Mannose derivative)

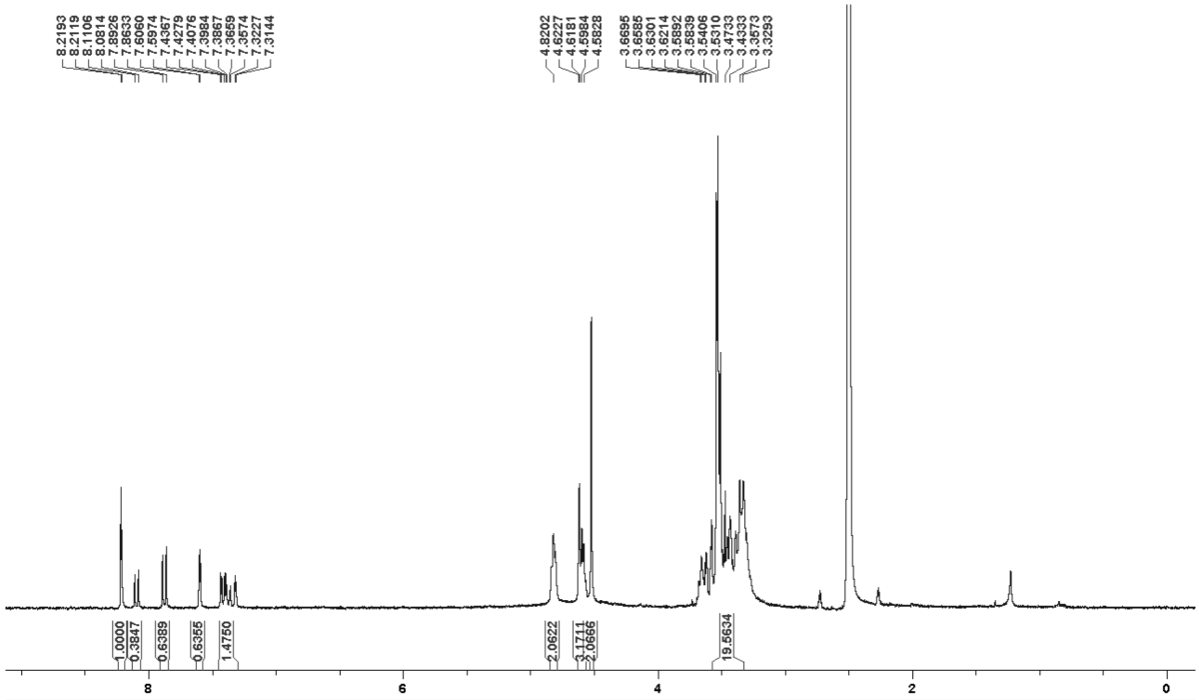

Supplementary Figure 23. ^{1}H **NMR spectra of compound 3**

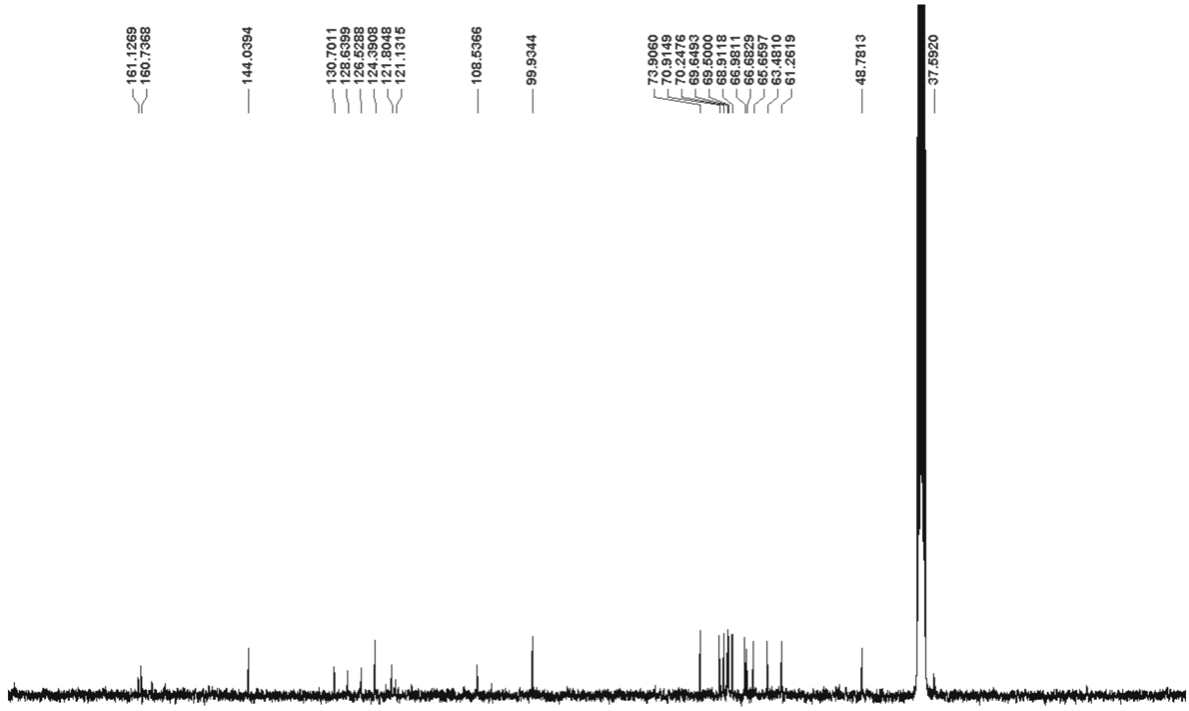

Supplementary Figure 24. ^{13}C **NMR spectra of compound 3**

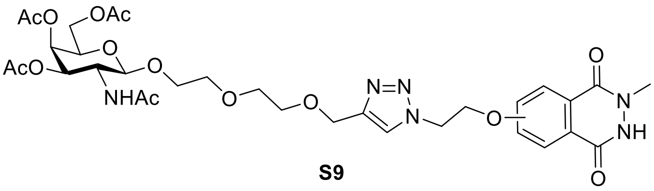

S9

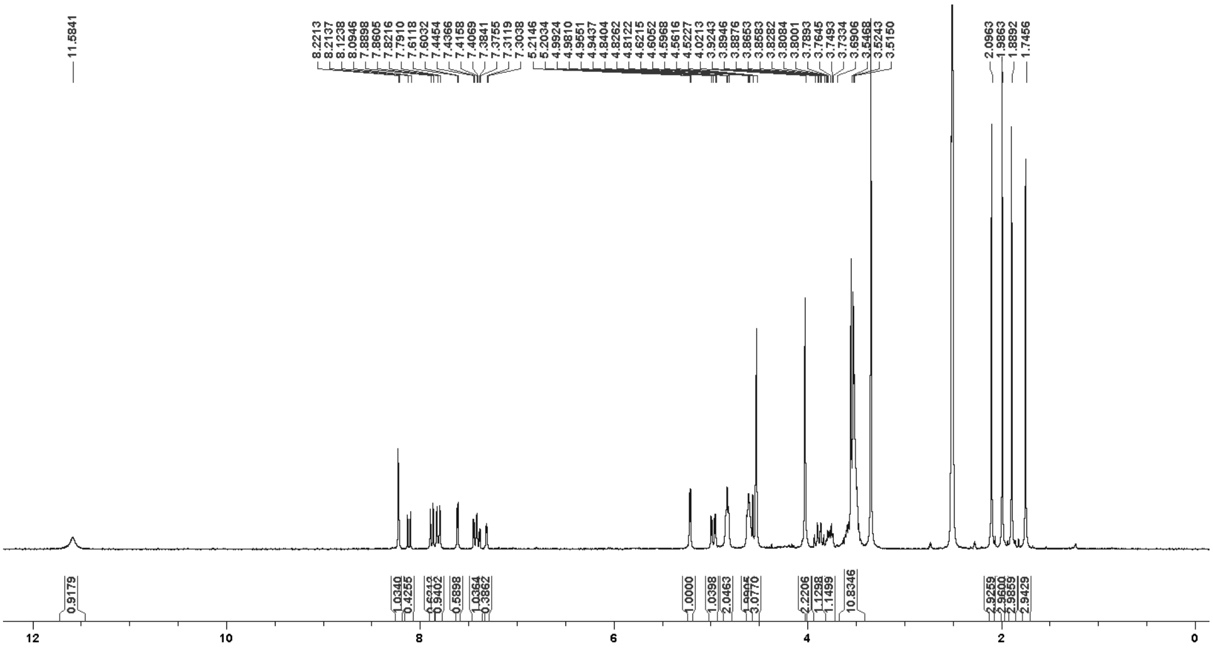

Supplementary Figure 25. ^{1}H NMR spectra of compound S9

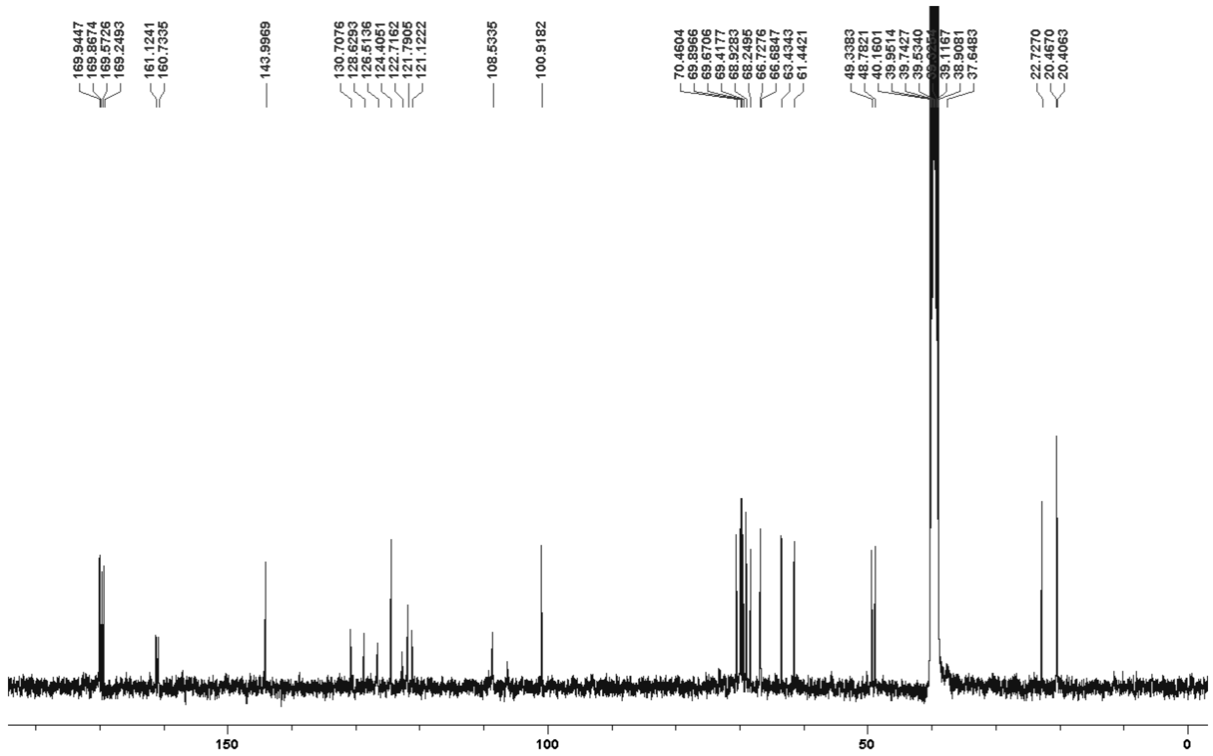

Supplementary Figure 26. ^{13}C NMR spectra of compound S9

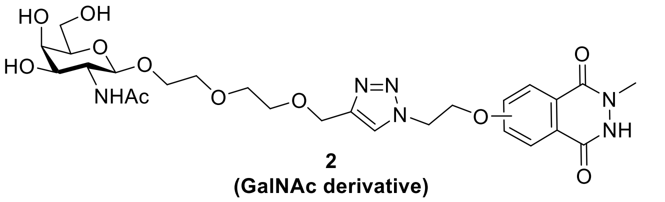

2
(GalNAc derivative)

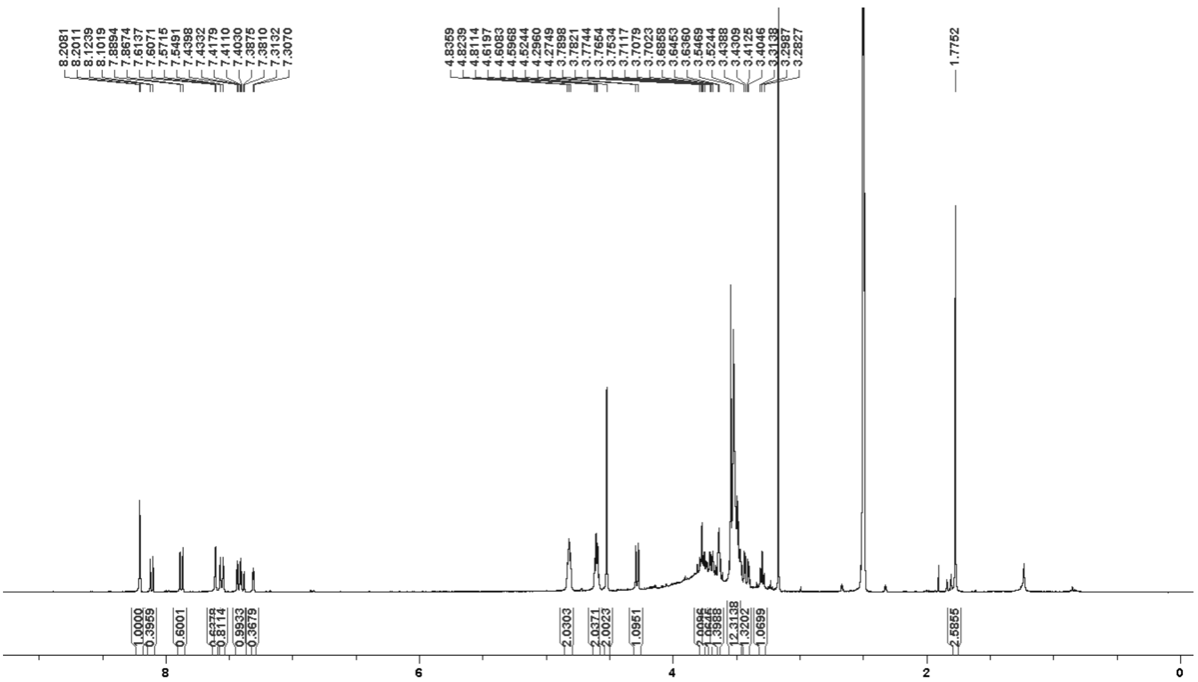

Supplementary Figure 27. ^{1}H NMR spectra of compound 2

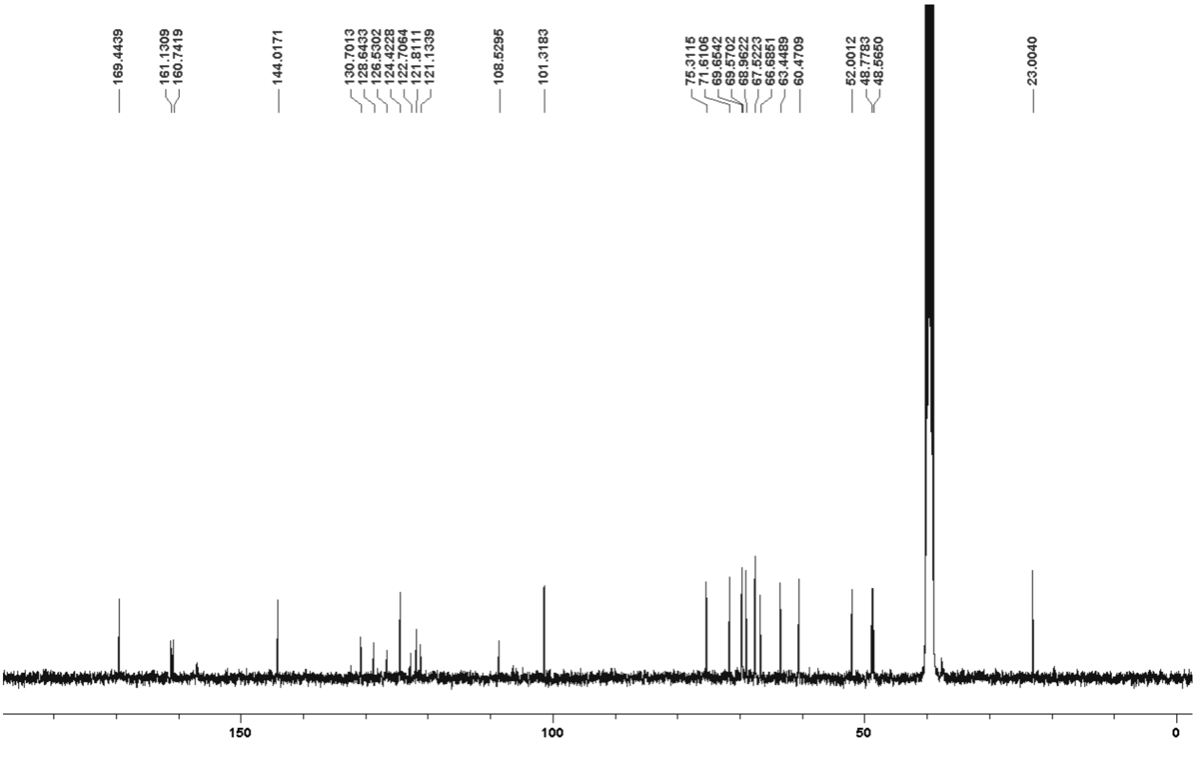

Supplementary Figure 28. ^{13}C NMR spectra of compound 2

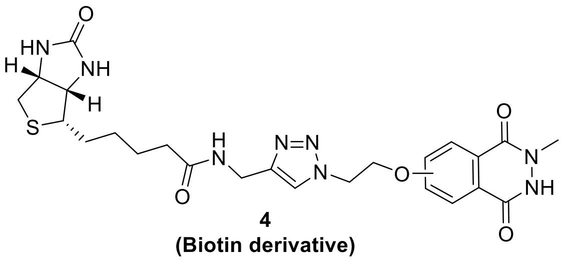

4
(Biotin derivative)

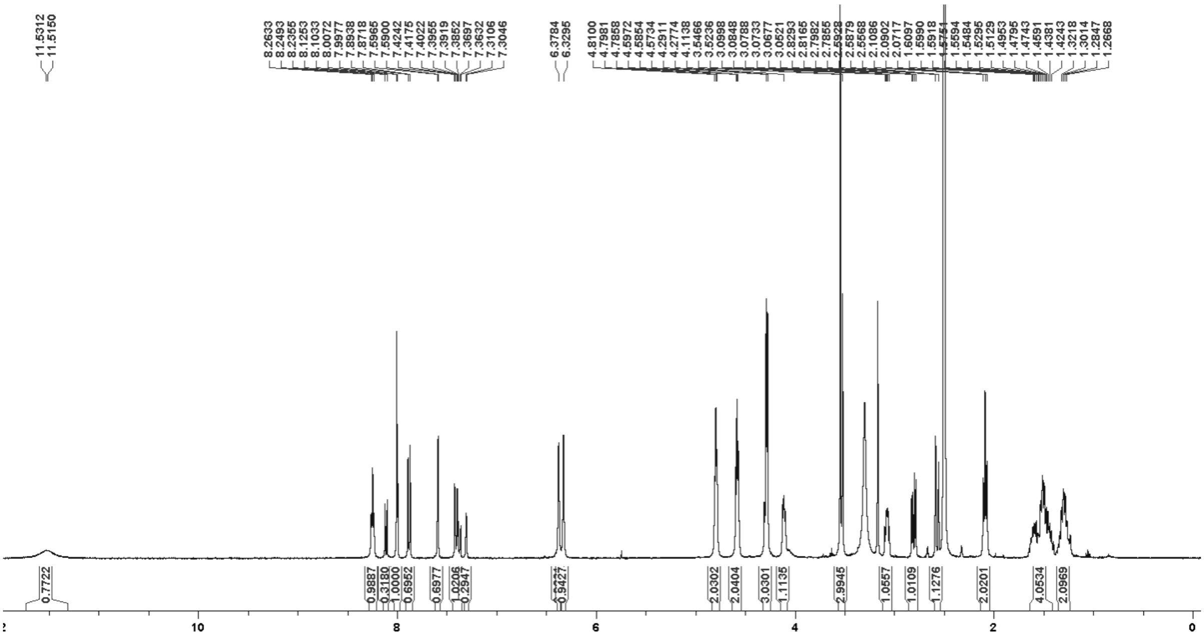

Supplementary Figure 29. ^{1}H NMR spectra of compound 4

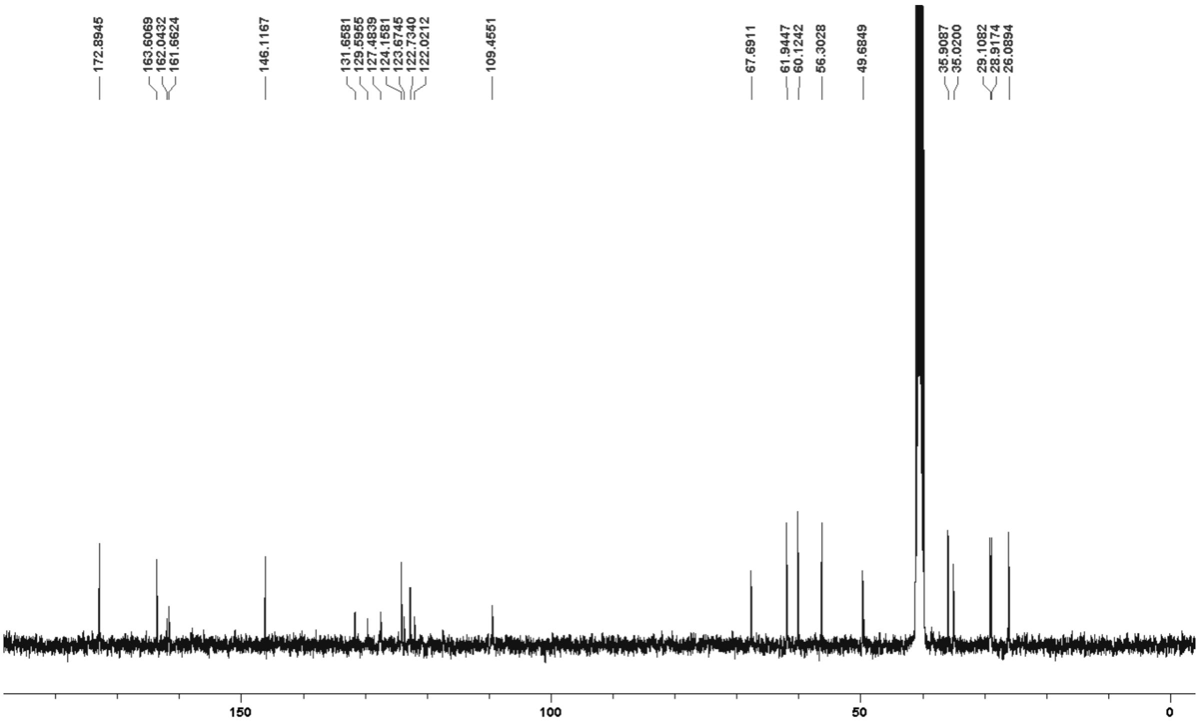

Supplementary Figure 30. ^{13}C NMR spectra of compound 4

VI. Uncropped western blots

Fig 2d

Left = Viral proteins detection (using anti-VPs polyclonal antibody) ; Right = mannose detection (using ConA lectin)
Here are the full gels where Fig 2d lanes are spotted with red arrows. Other lanes are samples from other projects irrelevant with the scope of this study
MW markers are not detected (VP and mannose detection) but were marked before revelation

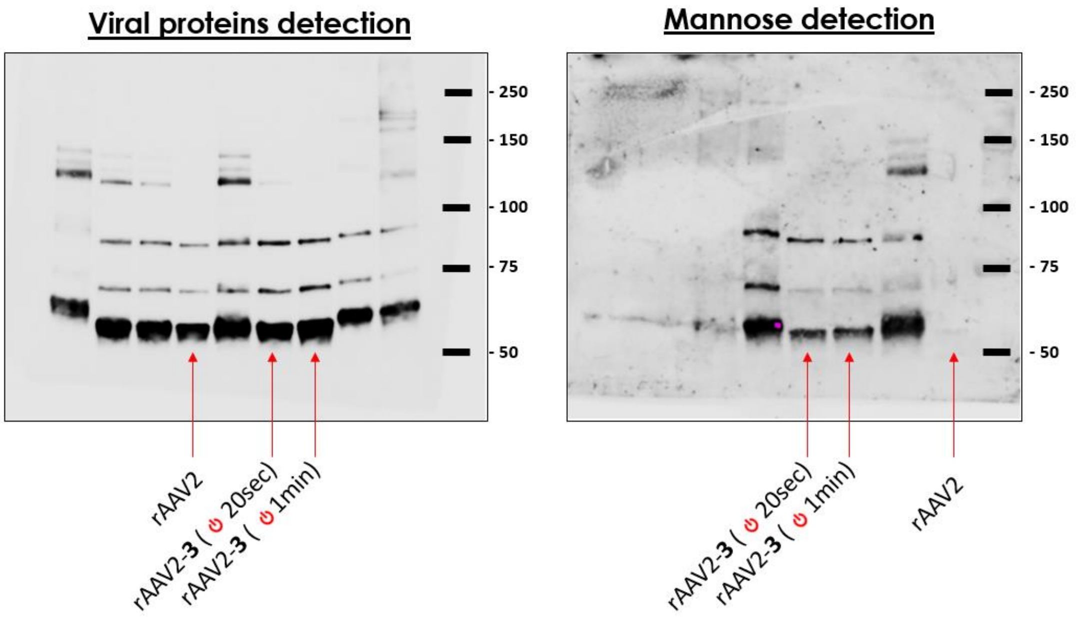

Fig 3e

Left = CBB revelation ; Right = fluorescence visualization at 490 nm
Here is the full gel with MW markers on the left and 2 replicates (2 different 60 min eY-click experiment on Ecoli) of lysis + SDS-PAGE analysis

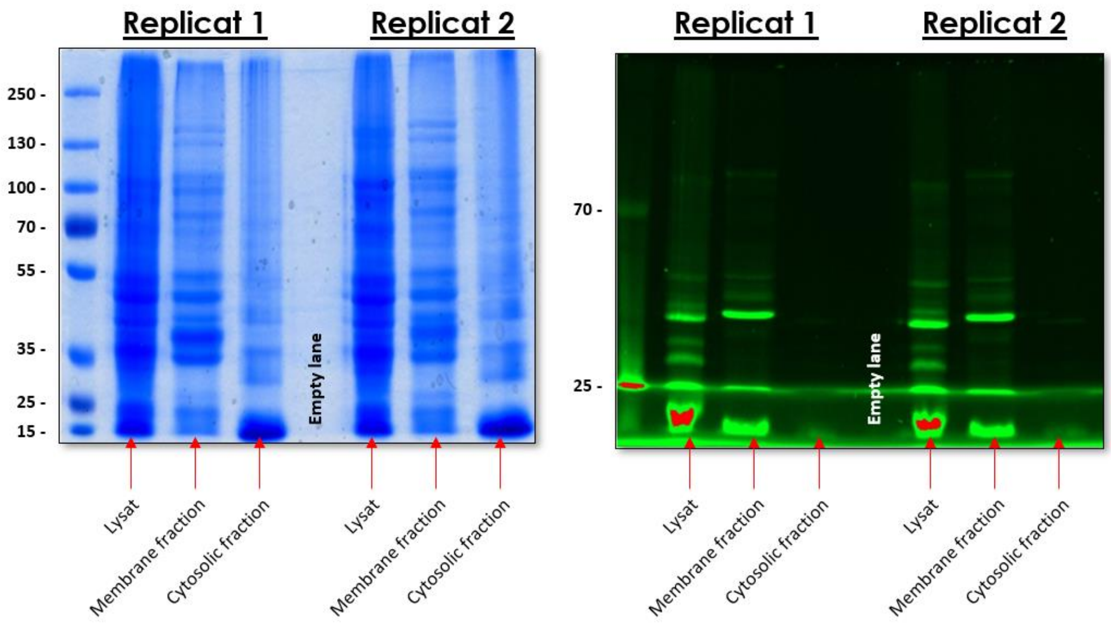

Fig S10a

Dot Blot with A20 antibody for assembled capsid integrity detection

Fig S10a dots are spotted with red arrows

Other dots are samples from other projects irrelevant with the scope of this study

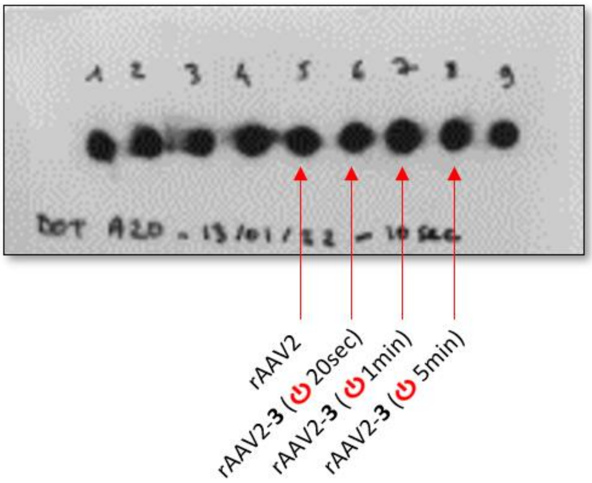

Fig S10b

Silver nitrate for all proteins detection (no dilution on MW markers, but these can still be identified)

Fig S10b lanes are spotted with red arrows

Other lanes are samples from other projects irrelevant with the scope of this study

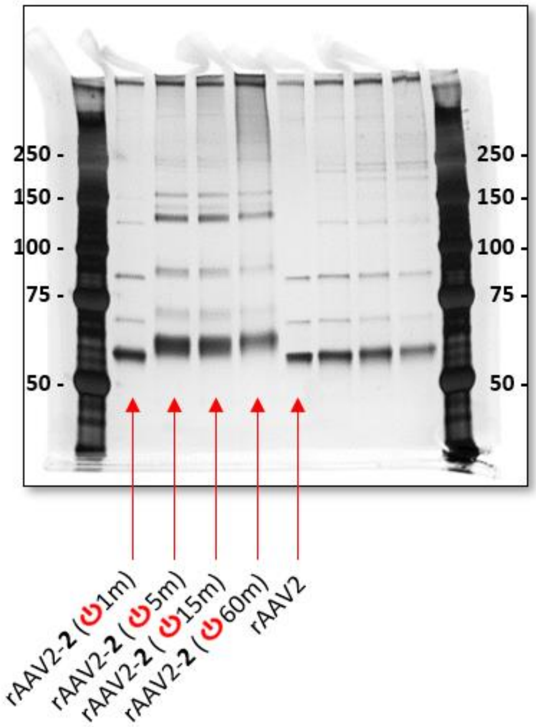

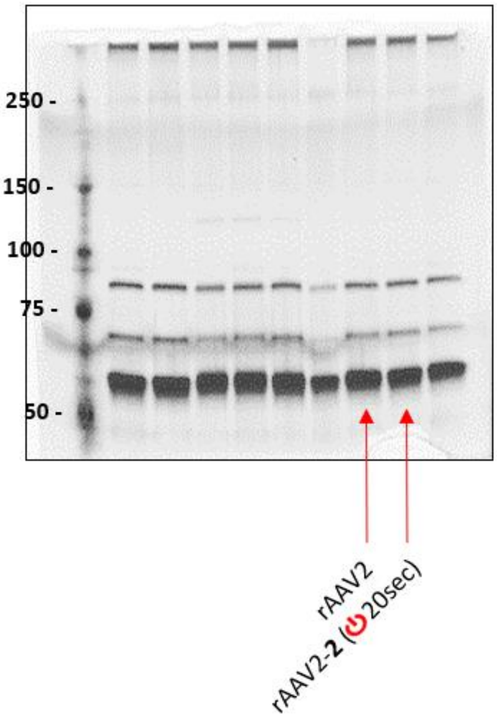

Fig S10c

Western blot with SBA-FITC to detect GalNAc

Fig S10c lanes are spotted with red arrows

Other lanes are samples from other projects

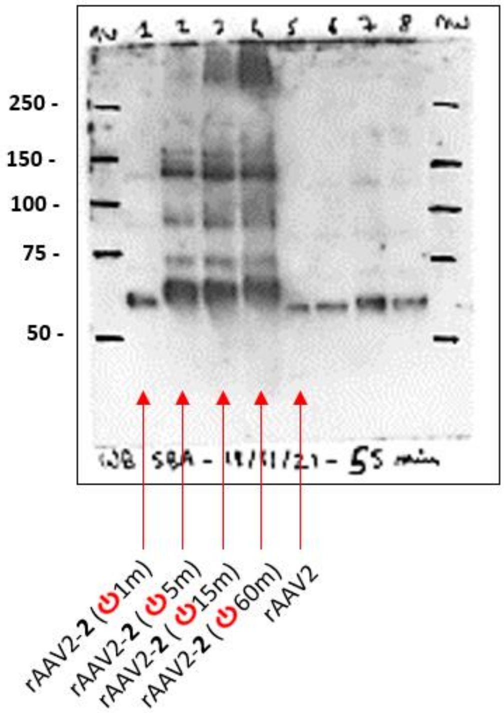

Fig S10d

Western blot with anti-VP polylconal to detect VPs

Fig S10d lanes are spotted with red arrows

Other lanes are samples from other projects irrelevant with the scope of this study

Viral proteins detection

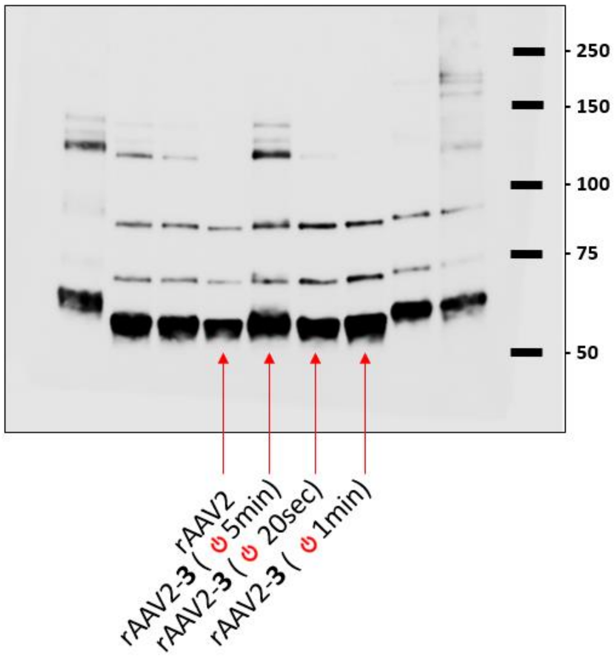

Fig S10e

Western blot with ConA-FITC lectin to detect mannose
Fig S10e lanes are spotted with red arrows
Other lanes are samples from other projects irrelevant with the scope of this study

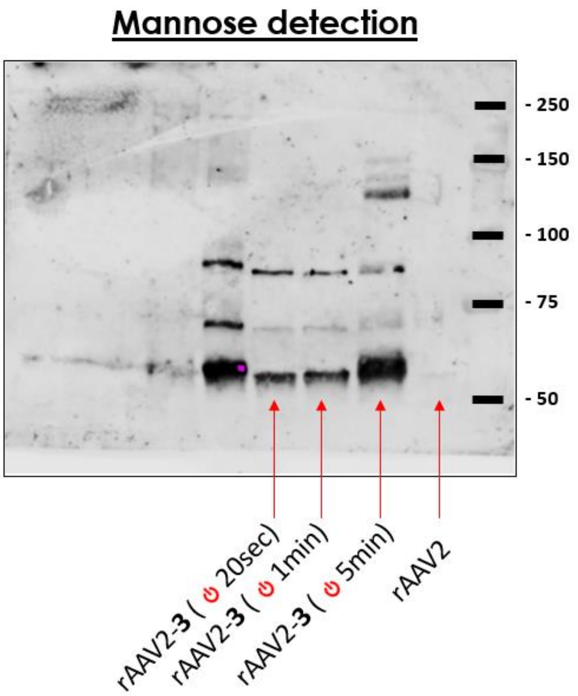

Fig S14

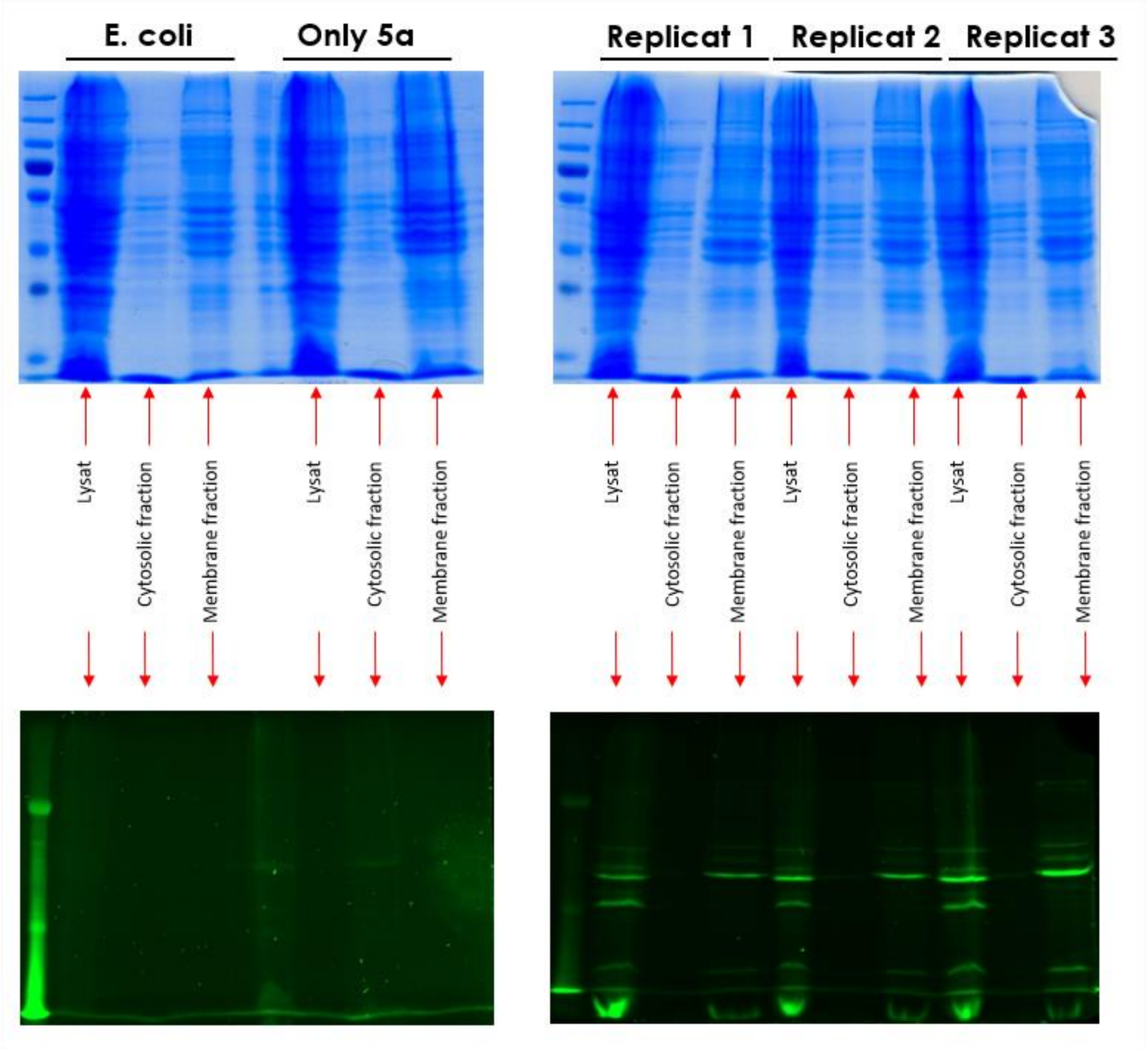
E. coli
Only 5a
Replicat 1
Replicat 2
Replicat 3
Lysat
Cytosolic fraction
Membrane fraction
Lysat
Cytosolic fraction
Membrane fraction
Lysat
Cytosolic fraction
Membrane fraction
Lysat
Cytosolic fraction
Membrane fraction
Lysat
Cytosolic fraction
Membrane fraction

Fig S15

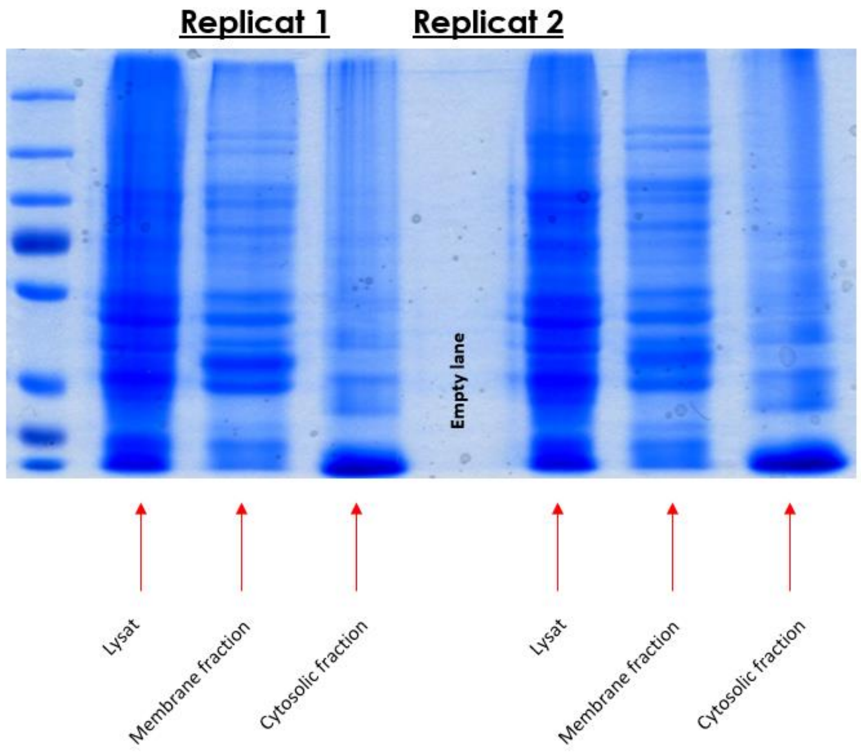

Replicat 1
Replicat 2
Empty lane
Lysat
Membrane fraction
Cytosolic fraction
Lysat
Membrane fraction
Cytosolic fraction

VII. Representative gating strategy.

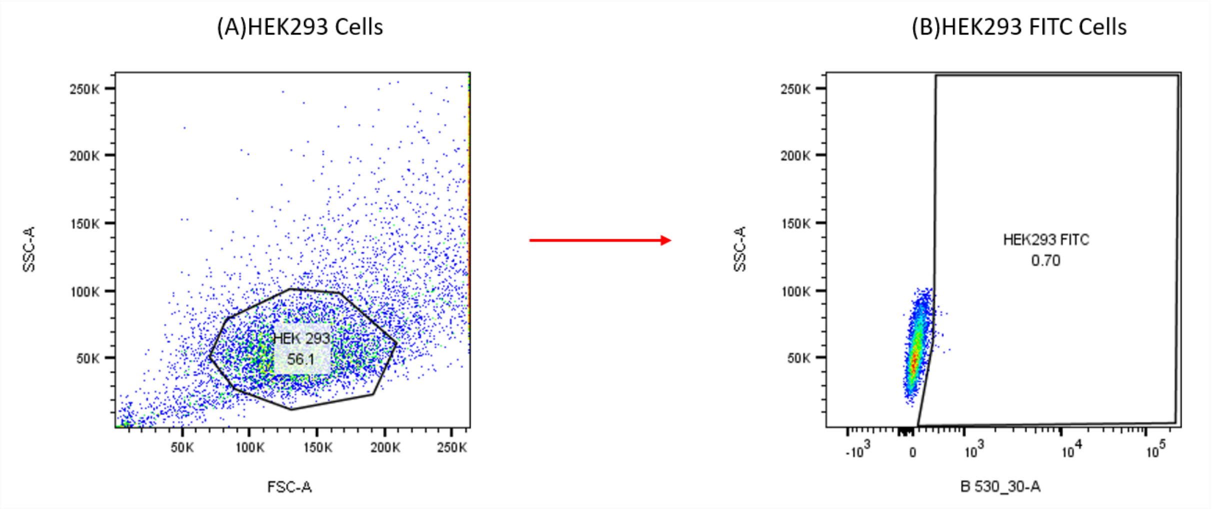

Supplementary Figure 31. Gating strategy for flow cytometry assay for HEK293 Cells. (A)FSC vs SSC gating was used to select HEK293 Cells. (B) B530_30-A were used to selected HEK293 FITC Cells.

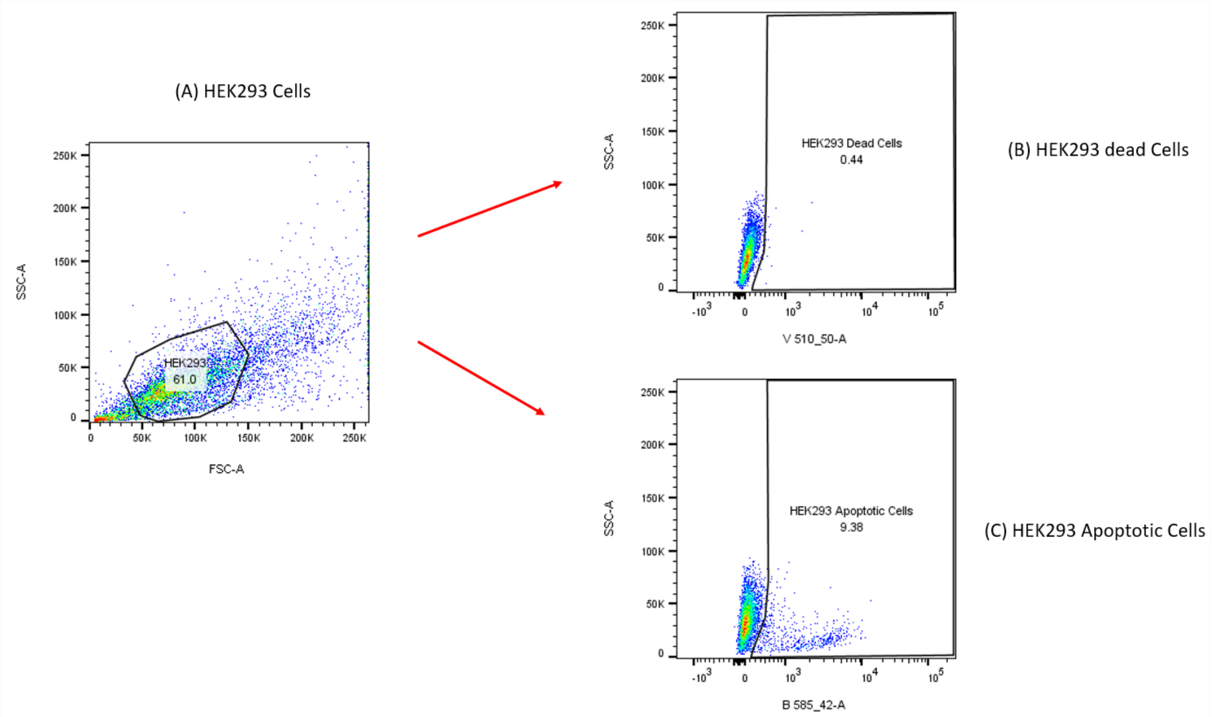

Supplementary Figure 32. Gating strategy for flow cytometry assay for HEK293 Cells. (A)FSC vs SSC gating was used to select HEK293 Cells. (B) 510-50A were used to selected HEK293 dead cells. (C) 585-42A were used to selected HEK293 Apoptotic cells.

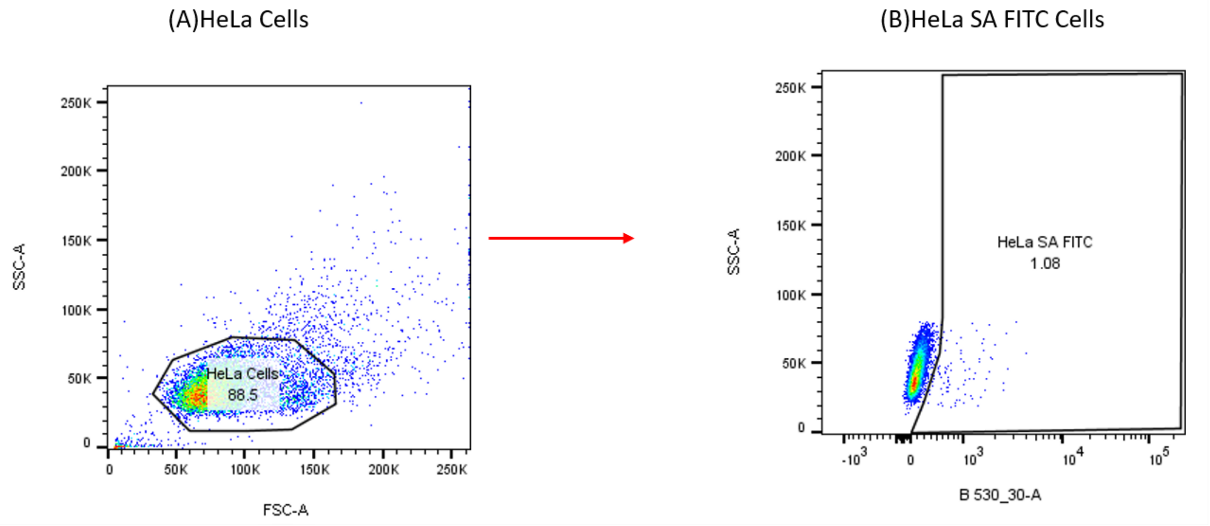

Supplementary Figure 33. Gating strategy for flow cytometry assay for HeLa Cells. (A)FSC vs SSC gating was used to select HeLa Cells. (B) B530_30-A were used to selected HeLa FITC Cells.

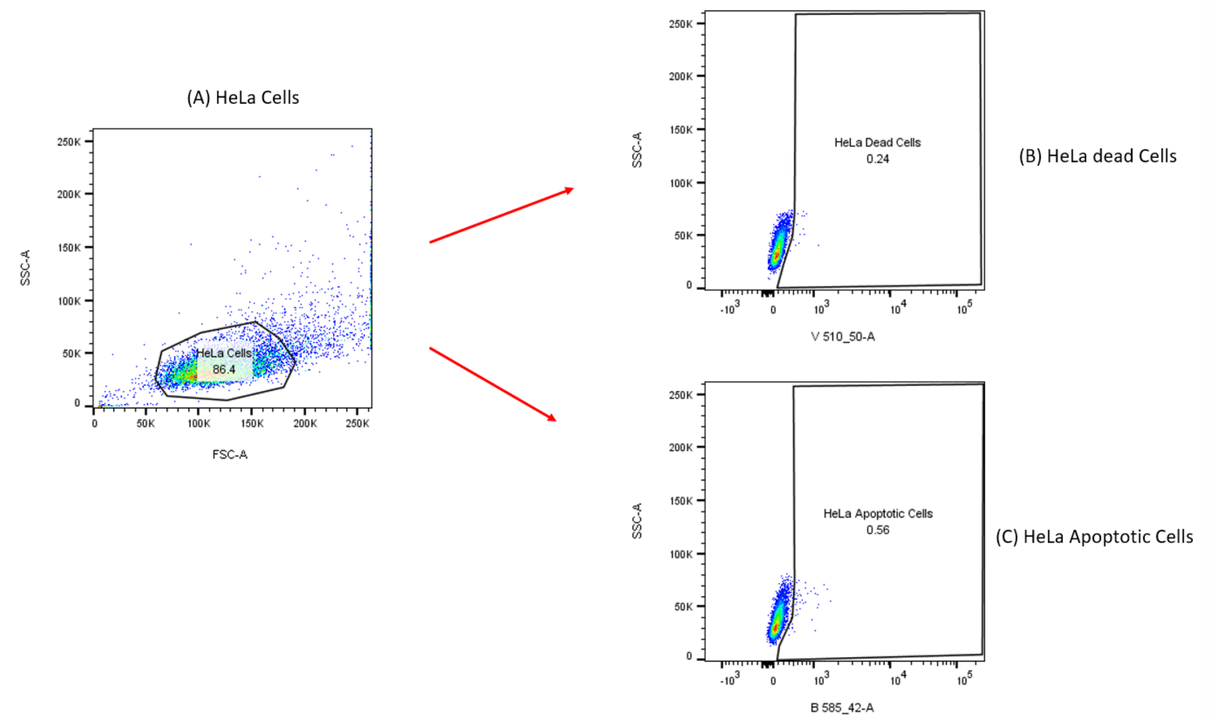

Supplementary Figure 34. Gating strategy for flow cytometry assay for HeLa Cells. (A)FSC vs SSC gating was used to select HeLa Cells. (B) 510-50A were used to selected HeLa dead cells. (C) 585-42A were used to selected HeLa Apoptotic cells.

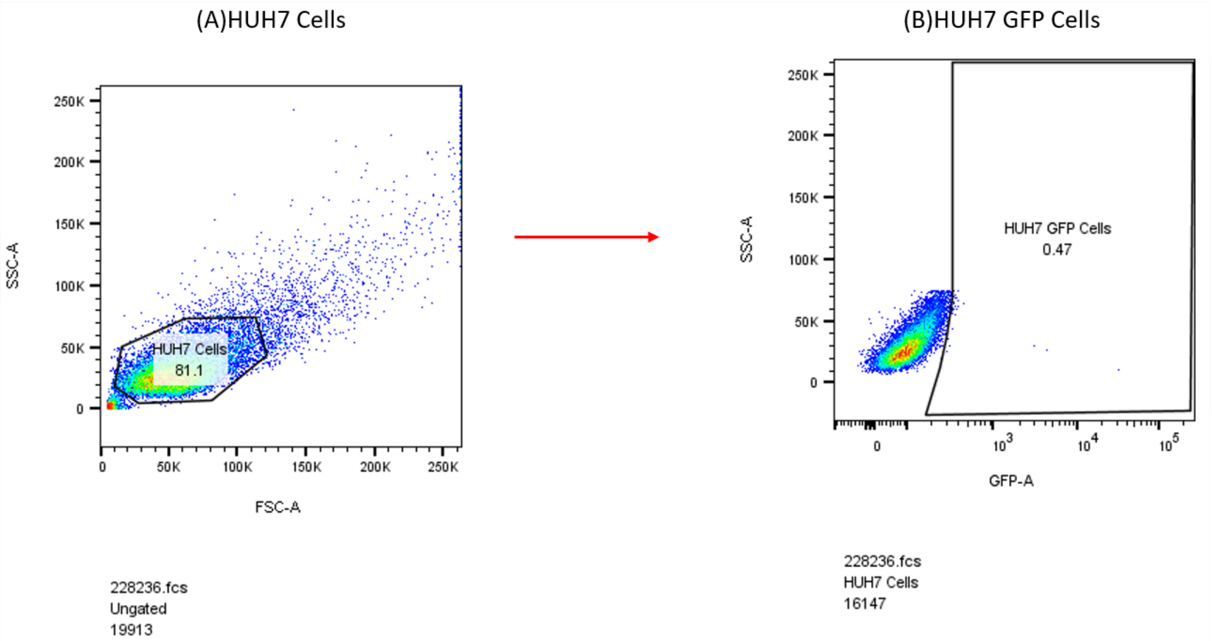

Supplementary Figure 35. Gating strategy for flow cytometry assay for HUH7 Cells infected with AAV vectors. (A)FSC vs SSC gating was used to select HUH7 Cells. (B) GFP-A were used to selected HUH7 GFP Cells.

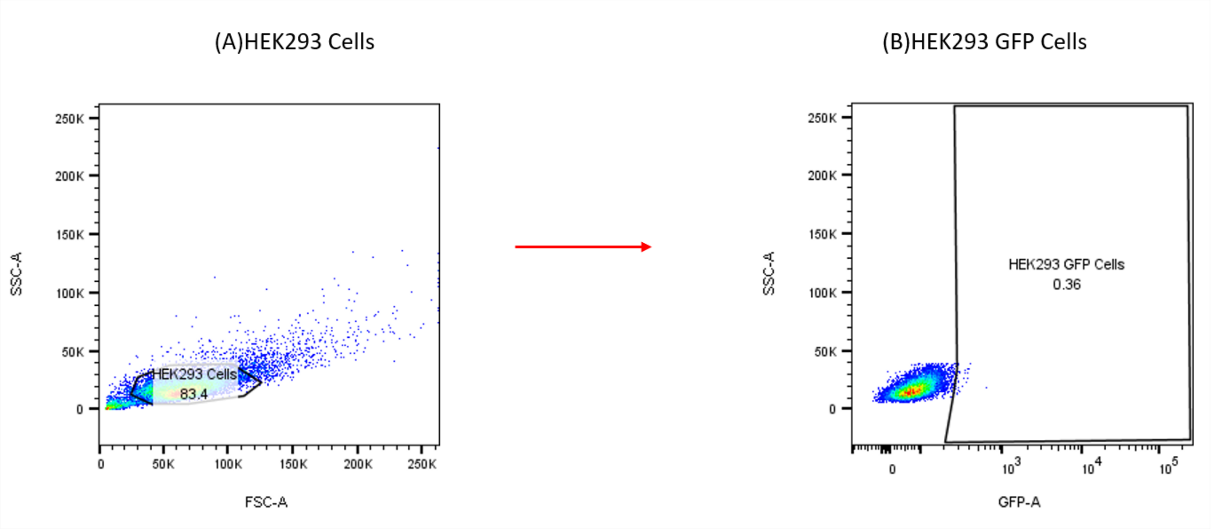

Supplementary Figure 36. Gating strategy for flow cytometry assay for HEK293 Cells infected with AAV vectors. (A)FSC vs SSC gating was used to select HEK293 Cells. (B) GFP-A were used to selected HEK293 GFP Cells.

VIII. Supplementary References

1. S. Depienne, *et al., Chem. Sci.,* **2021,** *12*, 15374-15381
2. C.-C. Lin, *et al.*, *Org. Lett.*, **2007**, *9*, 2131-2134
3. D'Costa et al., *Mol Ther Methods Clin Dev*., **2016**, *30*, 16019.
