# Supplementary material for: Click-electrochemistry for the rapid labeling of virus, bacteria and cell surfaces
Source: Nat Commun. 2023 Aug 23;14:5122. doi: 10.1038/s41467-023-40534-0 (PMC10447534; doi:10.1038/s41467-023-40534-0)
Supplement: Supplementary file 3 — Reporting Summary [file 41467_2023_40534_MOESM3_ESM.pdf]

nature portfolio

Corresponding author(s): Mathieu Mével, Sébastien Gouin

Last updated by author(s): Jul 7, 2023

Reporting Summary

Nature Portfolio wishes to improve the reproducibility of the work that we publish. This form provides structure for consistency and transparency in reporting. For further information on Nature Portfolio policies, see our Editorial Policies and the Editorial Policy Checklist.

Statistics

For all statistical analyses, confirm that the following items are present in the figure legend, table legend, main text, or Methods section.

n/a	Confirmed	
☐	☒	The exact sample size (n) for each experimental group/condition, given as a discrete number and unit of measurement
☐	☒	A statement on whether measurements were taken from distinct samples or whether the same sample was measured repeatedly
☐	☒	The statistical test(s) used AND whether they are one- or two-sided<br>*Only common tests should be described solely by name; describe more complex techniques in the Methods section.*
☒	☐	A description of all covariates tested
☐	☒	A description of any assumptions or corrections, such as tests of normality and adjustment for multiple comparisons
☐	☒	A full description of the statistical parameters including central tendency (e.g. means) or other basic estimates (e.g. regression coefficient) AND variation (e.g. standard deviation) or associated estimates of uncertainty (e.g. confidence intervals)
☐	☒	For null hypothesis testing, the test statistic (e.g. F, t, r) with confidence intervals, effect sizes, degrees of freedom and P value noted<br>*Give P values as exact values whenever suitable.*
☒	☐	For Bayesian analysis, information on the choice of priors and Markov chain Monte Carlo settings
☒	☐	For hierarchical and complex designs, identification of the appropriate level for tests and full reporting of outcomes
☒	☐	Estimates of effect sizes (e.g. Cohen's d, Pearson's r), indicating how they were calculated

Our web collection on statistics for biologists contains articles on many of the points above.

Software and code

Policy information about availability of computer code

Data collection	Data were collected with various instruments and equipements such as: Bruker Avance 400 spectrometer (NMR), Waters Xevo GL-XS Qtof spectrometer coupled with Acquity H-class LC apparatus (MS), CYTOFLEX S System flow cytometer Beckman Coulter or BD-LSDRII Flow Cytometer Diva 9, Infinite M1000 microplate reader TECAN (optical density measurment), Nikon Eclipse NI-E microscope: NIS-Elements AR 4.60.00, Nikon A1R microscope (confocal), ODYSSEY-CLX LI-COR imager (gel reader), Vi-CELL XR: XR2 (cells numeric data), Geldoc™EZ imager BIO-RAD (scanner).
Data analysis	EC-Lab V11.34 (electrochemical analysis and experiments), GraphPad Prism 8.0.1, FlowJo software V 10 (cytometry), CytExpert 2.4 Beckman Coulter (flow cytometer), NIS software: NIS-Elements AR 4.60.00 (microscopy), Magellan software V6.6 (optical density of bacterial samples), MassLynx V 4.0 (Mass Spectrometry), Software Image Studio Lite V 5.2 (gel reader), Image Lab™software version 5.2.1 BIO-RAD (scanner), Nikon Imaging Software NIS Elements AR 5.30.03 (confocal), TopSpin 4.0.7 (NMR).

For manuscripts utilizing custom algorithms or software that are central to the research but not yet described in published literature, software must be made available to editors and reviewers. We strongly encourage code deposition in a community repository (e.g. GitHub). See the Nature Portfolio guidelines for submitting code & software for further information.

Data

Policy information about availability of data

All manuscripts must include a data availability statement. This statement should provide the following information, where applicable:

- Accession codes, unique identifiers, or web links for publicly available datasets
- A description of any restrictions on data availability
- For clinical datasets or third party data, please ensure that the statement adheres to our policy

The data that support the findings of this study are available within the main text, Supplementary Information and Source data file

Research involving human participants, their data, or biological material

Policy information about studies with human participants or human data. See also policy information about sex, gender (identity/presentation), and sexual orientation and race, ethnicity and racism.

Reporting on sex and gender	N/A
Reporting on race, ethnicity, or other socially relevant groupings	N/A
Population characteristics	N/A
Recruitment	N/A
Ethics oversight	N/A

Note that full information on the approval of the study protocol must also be provided in the manuscript.

Field-specific reporting

Please select the one below that is the best fit for your research. If you are not sure, read the appropriate sections before making your selection.

[x] Life sciences [ ] Behavioural & social sciences [ ] Ecological, evolutionary & environmental sciences

For a reference copy of the document with all sections, see nature.com/documents/nr-reporting-summary-flat.pdf

Life sciences study design

All studies must disclose on these points even when the disclosure is negative.

Sample size	Triplicate from biologically independant samples was used to evaluate the repeatability of our experiments as it is classically done. The obtained data this sample size has not been increased as the variability coefficient (standard deviation / mean) is ≤ 20%. Confocal microscopy images (Fig 4b) were not repeated as the information obtained (membrane staining) is clear and unambiguous.
Data exclusions	No data were excluded from the analysis
Replication	Experiments were replicated three times (biological replicates), ensuring reproducibility. Replication attempts were successful, and precise numerical data were provided in the figure legends or tables for all experiments.
Randomization	In all experiments, samples were randomly assigned to different experimental groups.
Blinding	The investigators were blinded to the allocation of groups during both data collection and analysis

Reporting for specific materials, systems and methods

We require information from authors about some types of materials, experimental systems and methods used in many studies. Here, indicate whether each material, system or method listed is relevant to your study. If you are not sure if a list item applies to your research, read the appropriate section before selecting a response.

Materials & experimental systems

n/a	Involved in the study
☐	☒ Antibodies
☐	☒ Eukaryotic cell lines
☒	☐ Palaeontology and archaeology
☒	☐ Animals and other organisms
☒	☐ Clinical data
☒	☐ Dual use research of concern
☒	☐ Plants

Methods

n/a	Involved in the study
☒	☐ ChIP-seq
☐	☒ Flow cytometry
☒	☐ MRI-based neuroimaging

Antibodies

Antibodies used

Soybean Agglutinin-Fluorescein lectin from Vector Laboratories, Cat no FL-1011, Lot: ZH0409 used for Western bloting and Dot Blot.
Concanavalin A-Fluorescein lectin from Vector Laboratories, Cat no FL-1001-25, Lot: NA used for Western bloting and Dot Blot
antibody anti-Fluorescein-HRP from abcam, Cat no ab196968, Lot: GR3418767-6 used for Western bloting and Dot Blot
rabbit polyclonal anti-AAV capsid proteins from PROGEN Biotechnik, Cat no 61084, Lot: 798219-02 used for Western bloting
anti-rabbit-HRP from Jackson, Cat no 111-035-003
mouse anti-capsid A20 from Kleinschmidt Cat no: NA, Lot: NA
anti-mouse-HRP from Dako Cat no P0447, Lot : 41375555
Streptavidin-FITC solution from ThermoFisher, Cat no : SA1001, Lot: 2502894
Polyclonal anti-AAV capsid proteins has been modified by rabbit polyclonal anti-VPs antibody in the methods

Validation

All the lectins and the antibodies have already been validated and the data are published.
Mevel et al., Chemical Science, 2020, 11, 1122.
Mevel et al, BioRxiv, doi: https://doi.org/10.1101/2022.12.01.518481 .

Eukaryotic cell lines

Policy information about cell lines and Sex and Gender in Research

Cell line source(s)

HeLa is an immortalized cell line used in scientific research. The line is derived from cervical cancer cells, obtained from a research laboratory.
Human embryonic kidney 293 cells, are a specific immortalised cell line derived from human embryonic kidney cells, obtained from a research laboratory.
Huh7 is an immortalised cell line are differentiated hepatocyte-derived carcinoma cell line,obtained from a research laboratory.

Authentication

The three cell lines HEK293, HUH7 and HeLa are not recorded in the misidentified cell lines list. They were cultured based on ATCC protocol. The HEK293 cell line comes from our Working Cell Bank (WCB) number 3 batch 14/03/2016 used routinely for AAV vector production in our vector core (https://umr1089.univ-nantes.fr/en/facilities-cores/cpv). They are regularly characterized by qPCR and RT-qPCR for E1A gene expression (E1A 1411 forward primer: cacctcctgagtactccc; E1A 1546 reverse primer: cagctcaagtccaaaggtt and they are tested for mycoplasma free and sterility. The HUH7 and HeLa cell lines were kindly provided by academic laboratories and are regularly tested for mycoplasma free and sterility. These regular characterizations are performed under our ISO 9001 quality management system (LRQA certificate number 10395603, expiry date 30 September 2024).

Mycoplasma contamination

All cells are tested monthly for mycoplasma contamination. all tests were negative.

Commonly misidentified lines
(See ICLAC register)

There are no misidentified cell lines in this study

Flow Cytometry

Plots

Confirm that:

☒ The axis labels state the marker and fluorochrome used (e.g. CD4-FITC).

☒ The axis scales are clearly visible. Include numbers along axes only for bottom left plot of group (a 'group' is an analysis of identical markers).

☒ All plots are contour plots with outliers or pseudocolor plots.

☒ A numerical value for number of cells or percentage (with statistics) is provided.

Methodology

Sample preparation

All Cells samples were dissociated with Trypsin-EDTA (Sigma-Aldrich), washed with PBS 1X buffer (corning) and fixed with 4%

	paraformaldehyde (fisher scientific) and analysed on a BD-LSRII Flow Cytometer (BD Bioscience).
Instrument	BD-LSRII Flow Cytometer (BD Bioscience)
Software	FlowJo (V10, Flowjo LLC, Ashland, OR)
Cell population abundance	Assuming an abundance of 1.0 × 105P cells per samples
Gating strategy	The gating strategy used to analyze the responses of GFP positive cells or FITC are characterized by forward scatter (FSc) and side scatter(SSc), an acquisition gate was established based on FSs and SSc that included the cells but excluded most granulocytes and debris. 10,000 events were routinely collected to visualize and gate on this population.

☒ Tick this box to confirm that a figure exemplifying the gating strategy is provided in the Supplementary Information.
